# An Agent-Based Modelling Approach to Investigate the Impact of Sex and Gender on Tuberculosis Transmission: A Case Study of Kampala, Uganda

**DOI:** 10.1007/s11538-026-01734-z

**Published:** 2026-09-10

**Authors:** James W. G. Doran, Dennis Mujuni, Kit Gallagher, Christian A. Yates, Ruth Bowness

**Affiliations:** 1 Department of Mathematical Sciences, Centre for Mathematical Biology, University of Bath, Bath, BA2 7AY UK; 2 Department of Research and Development, Alpha Biomedical Research and Innovation Network, Kampala, Uganda; 3 Makerere University, College of Health Sciences, Kampala, Uganda; 4 Mathematical Institute, University of Oxford, Andrew Wiles Building, Radcliffe Observatory Quarter, Woodstock Road, OX2 6GG Oxford, UK; 5 Department of Pathology, Center for Cancer Research, Massachusetts General Hospital and Harvard Medical School, Boston, MA USA

**Keywords:** Tuberculosis, Agent-based model

## Abstract

Tuberculosis (TB) is an airborne disease caused by the pathogen *Mycobacterium tuberculosis*. In 2023, it returned to being the leading cause of death from an infectious agent globally, replacing COVID-19. More than 10 million people are diagnosed with TB every year. The majority of cases in adults occur in males (62.5% of all global adult cases in 2023, compared to 37.5% in females). The main reasons for males suffering from a higher burden of global TB cases, compared to females, may be in large part due to population-scale factors, such as employment type, the quantity and type of social contacts they make, and their health-seeking behaviours. To investigate which population-scale factors are most important in determining this higher TB burden in males, we have developed an age- and sex/gender-stratified, spatially heterogeneous epidemiological agent-based model. We have focused specifically on Kampala, the capital of Uganda, which is a high-burden TB country. We considered counterfactual scenarios to elucidate the impact of sex and gender on the epidemiology of TB, in order to deduce which factors have greater explanatory power in producing the observed differences between sexes/genders. Within-host parameters had the largest effect on overall case numbers among the scenarios considered. On the other hand, behavioural factors (particularly assortative mixing and gender-specific contact patterns) appear important in explaining the elevated male-to-female case ratio. We also found that super-spreaders appear to cause a majority of infections, with males and individuals with cavitary TB causing more infections on average. Our model provides a proof-of-concept framework that could help support public health policy after further validation.

## Introduction

Tuberculosis (TB) is an airborne disease caused by inhalation of the pathogen *Mycobacterium tuberculosis* (*M. tb*). According to the World Health Organisation, it ‘probably returned’ to being the leading cause of death from an infectious agent globally in 2023, having been replaced by COVID-19 for the previous three years (World Health Organization 2024b). In the nineteenth century, one in seven of all humans died of tuberculosis (Koch 1982); today, more than one million people die from the disease every year (World Health Organization 2025a). It is estimated that approximately a quarter of the world’s population are currently infected by the pathogen (Houben and Dodd 2016). In recent years, the majority of global incidence has occurred in high-burden TB countries: 86-90% of the world’s TB cases were distributed across thirty countries (World Health Organization 2024b). One of these countries is Uganda; we have focused on Kampala, the capital of Uganda, in this study. Although efforts are being made to detect TB hotspots and increase case finding in Uganda, using data-driven AI tools like EPCON (which has been effectively used in other countries, such as Nigeria (Alege et al. 2024)), it remains one of the worst affected nations, with an estimated 96,000 TB cases in 2023 (World Health Organization 2024a). Additionally, a greater number of TB cases in adults occur in males compared to females: in 2023, 62.5% of adults who developed TB were males, whilst 37.5% were females (World Health Organization 2024b).

The reasons for an increased TB burden in males compared to females could be in large part due to behavioural factors such as places of work, number of social contacts (Nhamoyebonde and Leslie 2014) and sex assortativity in adult contacts, given that like-with-like social mixing has been found to be commonplace for both males and females (Horton et al. 2020). Uganda is one of the thirty high-burden TB countries; recent studies by Nsawotebba et al. (2025) indicate that in Kampala, on average, males experience a longer delay than females in diagnosis. Gender has been found to be a factor in delays in diagnosis and treatment in Uganda in other studies as well (Buregyeya et al. 2014), and it has been observed that men are less likely to receive a timely diagnosis compared to women in low- and middle-income countries in general (Horton et al. 2016). This may be related to different health-seeking patterns between males and females and would provide evidence for the ‘behavioural hypothesis’ - one of two major hypotheses for the differences between genders in terms of TB prevalence - due to males being exposed to other males who have not yet been diagnosed. This is in agreement with evidence that suggests males in Uganda with less knowledge of the symptoms of TB typically have longer diagnosis delays (Obeagu 2023). The other major competing explanation is the ‘physiological hypothesis’, which suggests differences in immune response and genetics are responsible for the increased prevalence of TB in males, although the two hypotheses are not necessarily mutually exclusive (Nhamoyebonde and Leslie 2014). Mathematical modelling is a popular and versatile method to investigate whether TB transmission in Kampala is significantly influenced by gender, and in particular whether the differences in diagnosis delay have an impact on the male-to-female ratios of TB cases and deaths from TB.

Modellers can investigate the spread of tuberculosis using a number of different mathematical approaches. Here we use an agent-based modelling framework. Agent-based models (ABMs) explicitly model each individual within the population of interest; each of these ‘agents’ are assigned properties (such as age and gender), states (in the case of infectious disease models, these may include susceptible, infectious, recovered and others if appropriate) and rules to follow (who they are allowed to interact with, where to move if allowed - some examples could be moving up/down/left/right given a spatial location on a square lattice, or their location changing from ‘home’ to ‘work’ between time steps, and so on). Such a model simulates a disease outbreak by allowing these agents to interact with other agents and/or the environment, and transmit, or be infected by, the pathogen being investigated. Some previous epidemiological models of TB have used agent-based modelling frameworks (e.g. de Espíndola et al. (2011); Guzzetta et al. (2011); Tian et al. (2013); Kasaie et al. (2015); Prats et al. (2016); Shrestha et al. (2017); Ragonnet et al. (2019); Zwick et al. (2021); Udall and Searle (2023); see Bui et al. (2024) for a systematic review). Additionally, other TB transmission models have explored the impact of gender on the likelihood of developing active TB and dying from the disease (e.g. Kisselevskaya-Babinina et al. (2018); Kubjane et al. (2023); Wang and Cao (2024); Richards et al. (2025)). However, to our knowledge, no previous agent-based model has explored gender-differentiated TB transmission in a high-burden setting, such as Kampala, so our approach will fill a gap in existing research by combining gender/sex stratification with an agent-based modelling framework to study TB in a high-burden setting.

In this paper, we adapt an agent-based model, Epiabm (Gallagher et al. 2024), which was a re-implementation of CovidSim (see Ferguson et al. (2020) and Appendix A for more details); the adapted version of Epiabm that we use throughout the rest of this paper will be referred to as EpiabmTB. Firstly, we have altered some of the features of the Epiabm framework to make it more specific to TB (for example, vaccination - from queued vaccination according to priority for COVID-19 to assuming the majority of individuals have already been vaccinated and determining whether newborns are vaccinated according to some probability for TB - and treatment using antimicrobials - from antivirals for COVID-19 to antibiotics for TB). Secondly, we have added in new elements (presented in Sect. 2.2) to make the model more capable of capturing the epidemiological dynamics of TB that are not as relevant to COVID-19.

We fit the model outlined in this paper to case count and death count data for a scaled-down version of the population of Kampala, Uganda to validate our approach, before using the model to explore counterfactual scenarios and observe their impact on the male-to-female ratio of TB cases and deaths. In this way, we can investigate the importance of different ‘between-host’ factors (such as differences in diagnosis delays between genders, the proportions of each gender in schools/workplaces and sex assortativity of contacts) and ‘within-host’ factors (such as differences in the probability of progression from latent TB to active TB and the probability of developing cavitary TB) in determining what contributes to the high TB burden observed in males. We can therefore provide evidence for the behavioural hypothesis or the physiological hypothesis, by deducing which factors have greater explanatory power in producing the observed differences between genders.

The rest of this paper is structured as follows. In Sect. 2, we provide a brief overview of the alterations we have made to the Epiabm model in order to represent TB in Kampala with an age- and gender-stratified population. In Sect. 3, we present the results generated using our model, comparing the calibrated data against counterfactual scenarios where differences between males and females are removed. In Sect. 4, we discuss the implications of our work and planned future work following on from this study.

## Methods

In this section, we give a brief overview of Epiabm - the previously developed agent-based model upon which our model is based - before discussing the addition, removal and alteration of features we have made to adapt the Epiabm model specifically towards modelling TB epidemiological dynamics, and then describing the parameter estimation for our model. In Appendix A, we provide a more detailed overview of Epiabm.

### Epiabm

Epiabm is a spatial agent-based model designed to simulate infectious disease spread across a geographic region. Individuals are placed on a grid of cells subdivided into microcells, with households, schools, and workplaces allocated based on population density. Each person is assigned demographic attributes and an infection state drawn from standard compartments (Susceptible, Exposed, Infectious, Recovered, Removed), with infectious states further categorised by severity. Transmission occurs through household contacts, place-based interactions, and random social meetings, with probabilities influenced by distance via spatial kernels. The model initialises with most individuals susceptible and a small number infected, and infections propagate according to calculated forces of infection. Both Python and C++ backends are provided, as well as an object-oriented structure for population generation and storage. It is easy to adapt and extend the model, although this comes at the expense of reduced computational efficiency. The model allows for population initialisation from density files or random placement, assignment of household structures and ages using heuristic algorithms, and allocation of schools and workplaces based on distance-weighted probabilities. Infection progression follows user-specified distributions, and transmission dynamics are updated at each time step, accounting for household, place, and inter-cell interactions.

We exploit the flexible framework provided by Epiabm for exploring epidemic dynamics in spatially structured populations, enabling detailed representation of demographic heterogeneity and contact patterns.

### Alterations

We have altered Epiabm to be more applicable to TB in a number of key aspects. We have redefined the disease states individuals can have at any point in time, as individuals can be carriers of latent TB infection for years after their initial exposure to *M. tb* and can relapse even after successful treatment. In addition, we have added the ability of individuals to be born, age, die of non-TB causes, and move (i.e. be re-assigned to new) households and places; this evolution of the socio-demography has been added to take into account the fact that the serial interval is typically longer for TB relative to COVID-19. To investigate the role of gender on transmission dynamics, we have introduced gender as an attribute of individuals within the model, with separate proportions of each gender in each place type, the consideration of genders in household allocation, and separate diagnosis delay probability distributions.

Additional details about these alterations are given in Sects. 2.2.1-2.2.5, where we implement them and discuss the impact they have on the model results.

#### Disease States

We have chosen the potential disease states an individual can have at any given point in time based upon those used by Prats et al. (2016). In total, there are 11 potential states. Newborn individuals initially enter the ‘Healthy’ compartment (identical to the ‘Susceptible’ compartment referred to in Appendix section A.1).

If a healthy individual comes into contact with an individual currently experiencing active TB, there is the possibility that they are exposed to the *M. tb* pathogen. If they are, they transition to the ‘Latent TB’ compartment (this is identical to the ‘Exposed’ compartment referred to in Appendix sections A.1 and A.3). If they recover naturally (that is, their immune response is able to completely remove all *M*. *tb* pathogens without the need for antibiotics), they will transition back to the healthy compartment after the period of time it takes the individual to recover naturally.

Alternatively, if individuals in the ‘Latent TB’ compartment become sick, they transition to the ‘Active TB’ compartment. This is identical to the ‘Infectious’ compartment referred to in Appendix section A.1, but is not sub-divided into categories based upon severity and the presence or absence of symptoms; it is assumed all individuals with active TB have equally severe disease and are equally symptomatic. It is worth noting that the Infectious compartment was sub-divided when compartmentalising COVID-19 infection states to help track hospitalisations and ICU visits more easily, which is not an aim of this paper, so the impact of not subdividing the infectious compartments on the model output should be negligible in this regard. Individuals in this compartment are capable of transmitting the disease to other healthy individuals. This can happen immediately after transitioning between the ‘Healthy’ and ‘Latent TB’ compartments (that is, the individual transitions from ‘Healthy’ to ‘Latent TB’ to ‘Active TB’ in the same time step), according to the ‘fast TB’ probability. Otherwise, it occurs after an exponentially distributed period of time, with this ‘slow TB’ probability equal to one minus the fast TB probability.

A literature review of how progression from latent TB to active TB has previously been mathematically modelled was conducted by Menzies et al. (2018). Based on that review, our model structure - with a single latent compartment and the possibility to transition straight from Healthy to Active TB - is closest to Structure E (see Appendix B for an overview of each structure considered in Menzies et al. (2018)). In a similar literature review, Ragonnet et al. (2017) found Structure E to be the worst or second-worst performing model structure for capturing progression from latent TB to active TB; having two latent compartments led to a better fit. However, due to limited data availability to inform parameter selection for the more complicated structures outlined in Appendix B, including those with two latent compartments, we have chosen to implement this structure. Our model structure corrects for the issue present in some common model structures which don’t allow for an immediate decline in progression risk after infection. Unlike any of the structures explored in Menzies et al. (2018), our model structure allows for natural recovery from infection and a return to the ‘Healthy’ compartment, which should compensate for the fact Structure E typically overestimates long-term progression risk.

Infectiousness of individuals in the ‘Active TB’ compartment is dependent on age; older individuals are more infectious than younger individuals (Ragonnet et al. 2019). In addition, with a certain probability, individuals can develop cavitary tuberculosis, with cavitations forming in their lungs, which increases infectiousness by a scalar multiplier. Males have a higher probability of developing cavitary TB than females (Balogun et al. 2021), which is reflected in the probabilities of having cavitary TB according to gender in our model. We have assumed that the infectiousness of individuals does not change over the course of their infection.

If the individual is diagnosed as having active TB, they transition to the ‘Under treatment’ compartment; they start receiving treatment and stop being infectious at this point. This is a valid assumption since evidence suggests effective treatment can reduce the transcription of genes associated with TB infectiousness after one day of treatment (Shaikh et al. 2021). Each gender has a separate diagnosis delay distribution when transitioning from the ‘Active TB’ compartment to the ‘Under treatment’ compartment, reflecting the fact that gender appears to impact the delay between developing active TB and being diagnosed. From here, the individual receiving treatment may stop treatment, and subsequently relapse to the ‘Active TB’ compartment; otherwise they will transition to the ‘Recovered but could relapse’ compartment upon successful completion of their treatment.

**Fig. 1:**
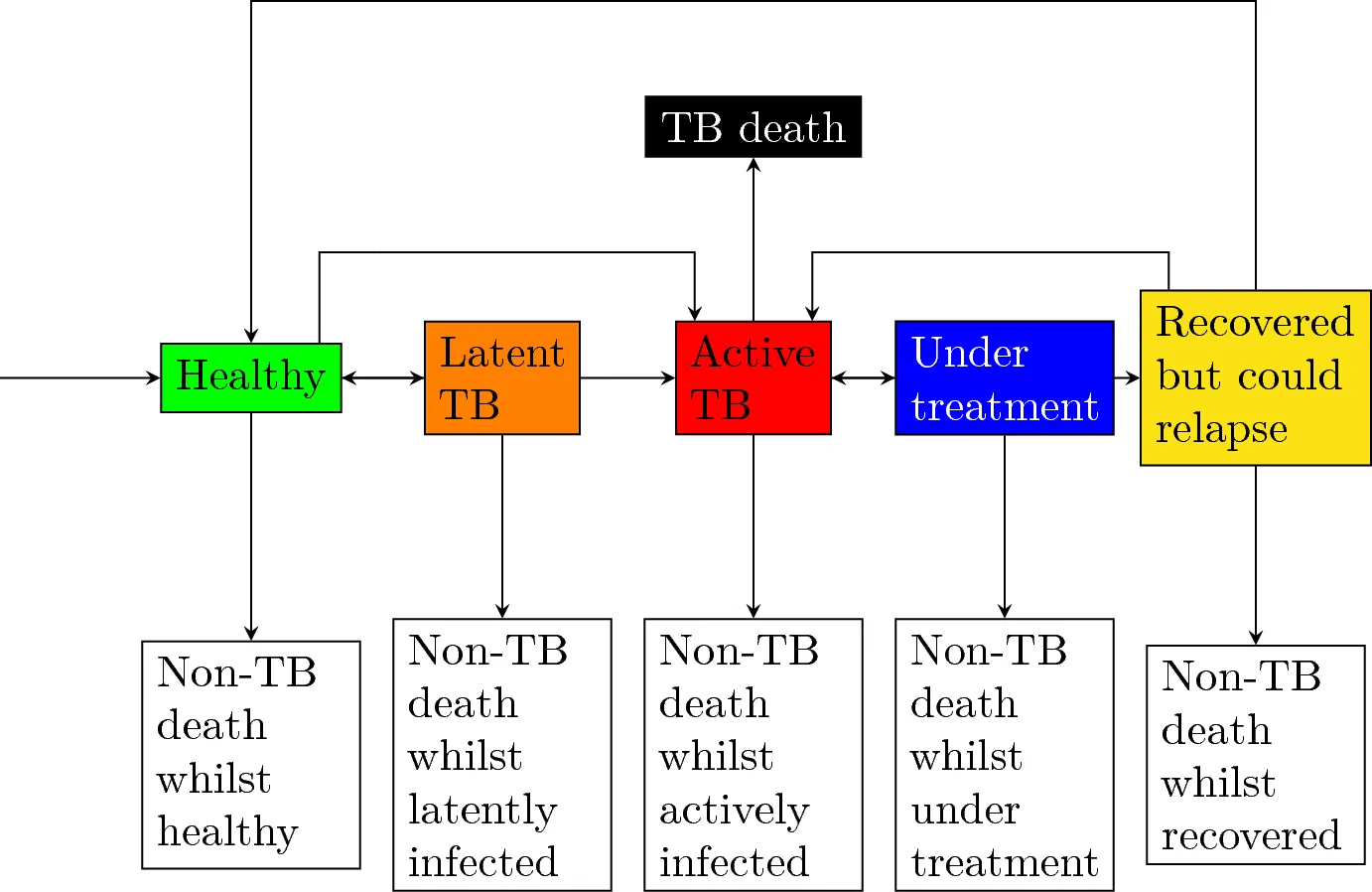
Infectious disease states used in our TB epidemiological model and the potential transitions between them. The arrow into the Healthy compartment from the left of the figure represents births: newborns always enter the Healthy compartment at birth. See text for full description

Individuals in the ‘Recovered but could relapse’ state can relapse and return to the ‘Active TB’ compartment, or transition back to the ‘Healthy’ compartment once their risk of relapse is zero. For every one of the five non-newborn compartments previously mentioned, there is a compartment for those who die whilst in one of those states for reasons other than TB. Additionally, an individual experiencing active TB can die from TB and transition to the ‘TB death’ compartment. We have assumed that once an individual starts treatment, their increased risk of dying from TB is removed, in keeping with Prats et al. (2016). A schematic of the compartments and the transitions between them can be seen in Fig. 1.

#### Evolution of Socio-Demography

The dynamics of TB infection play out over a longer period of time than those of COVID-19, which Epiabm was originally designed to model. As such, we have added in the possibility for new individuals to be born, for individuals to age and potentially die of non-TB causes, and for individuals to move households and places. Our methodology is adapted from Guzzetta et al. (2011). Births and non-TB deaths take place once per simulated day in our model, and ageing and relocation of households and places takes place once per simulated year in our model to reduce computational cost.

A new individual can be born into a household, provided the household has an adult female of suitable age already residing there who hasn’t given birth within the previous nine months. For the parameter values used for age allocation within households, please refer to Table 5 in Appendix C.

Individuals increase their age by one every year, and move into the next 5-year age group if old enough to do so. Individuals can either die from non-TB causes with a certain probability every day, or will do so automatically once they reach the maximum modelled age of 100, which we have assumed is the oldest age of individuals in the population based on data from United Nations (2024a). This seems a reasonable assumption, as although approximately 1.1% of the Ugandan population are 80 years old or older (Uganda Bureau of Statistics 2024), fewer than 0.01% of Ugandans are 100 years old or older (United Nations 2024a). The date at which 100-year-olds are removed is chosen uniformly at random between the day they turn 100 and 364 days later, including both endpoints of this range, to increase the realism of the model. Due to insufficient data on social contacts for older individuals from Prem et al. (2017), we have assumed all individuals aged 75 and older have the same contact rates.

There are two ways of moving household in our model: children becoming adults and moving into their own households, and married adults divorcing and moving into a single-person household. An individual that just became an adult and moves out from their original household, referred to as adult A for clarity, can partner up with somebody of a suitable age from the opposite gender; this couple then forms a new household in the same cell as adult A. We have made the assumption that in every household with a couple, the couple consists of one male and one female; this seems justified given that the Ugandan parliament passed an ‘anti-homosexuality’ bill in 2023 (Kakumba 2023) and related laws formalised in the country since the 1902 Order in Council, when Uganda was a British protectorate (Jjuuko 2013). Polygamy is legal and not uncommon in Uganda (Mwanga et al. 2021), so we have allowed for the possibility of husbands having a second wife in the same household. Whether or not a man has a second wife is determined by comparing the percentage of men with more than one wife to a number chosen uniformly at random between 0 and 1. We have assumed no man has more than two wives. In the case of divorce, one of the divorcees relocates to a new household within the same cell as the original household.

We have considered two place types in our model: schools and workplaces. This is different to Epiabm, which splits schools into primary, secondary and sixth form. The reduction in the number of types of schools is justified by the fact that children are rarely the source of tuberculosis transmission (Logitharajah and Kampmann 2008). We have also removed care homes and outdoor spaces as place types; we have taken this step to simplify the model. Within each place type, we have two age groups, each with an associated proportion of the population belonging to that age group and gender which attend that place type (e.g. pupils and teachers in school). Once an individual ages beyond the maximum age of the age group they previously belonged to for a certain place type (e.g. once a pupil in school becomes too old to remain a student), they are removed from that place. They may then be assigned somewhere new, based on the proportion of individuals of their age and gender for the place type of the new place being proposed.

#### Gender Stratification

Each individual is assigned a gender with probability drawn from a probability distribution determined according to gender distribution data in Uganda (see Table 5 in Appendix C). In this model, we have assumed individuals are assigned one of two sexes at birth and identify as belonging to one of two genders. In addition, we have assumed each individual’s gender identity aligns with their assigned biological sex, that is, individuals assigned the male sex at birth identify as men/boys and individuals assigned the female sex at birth identify as women/girls. It should be noted that any biological ‘within-host’ parameters (such as cavitation probability, probability of progression from Latent TB to Active TB, and TB mortality) are sex-linked, while any behavioural ‘between-host’ parameters (such as diagnosis delay, workplace participation, and contact patterns) are gender-linked. Gender is considered along with age when distributing the population between the microcells, and when assigning individuals to places, during initialisation of the model: pupil-age females are more likely to be in school than pupil-age males, but adult-age males are more likely to be in workplaces than adult-age females, thereby capturing differences in workplace participation.

**Fig. 2:**
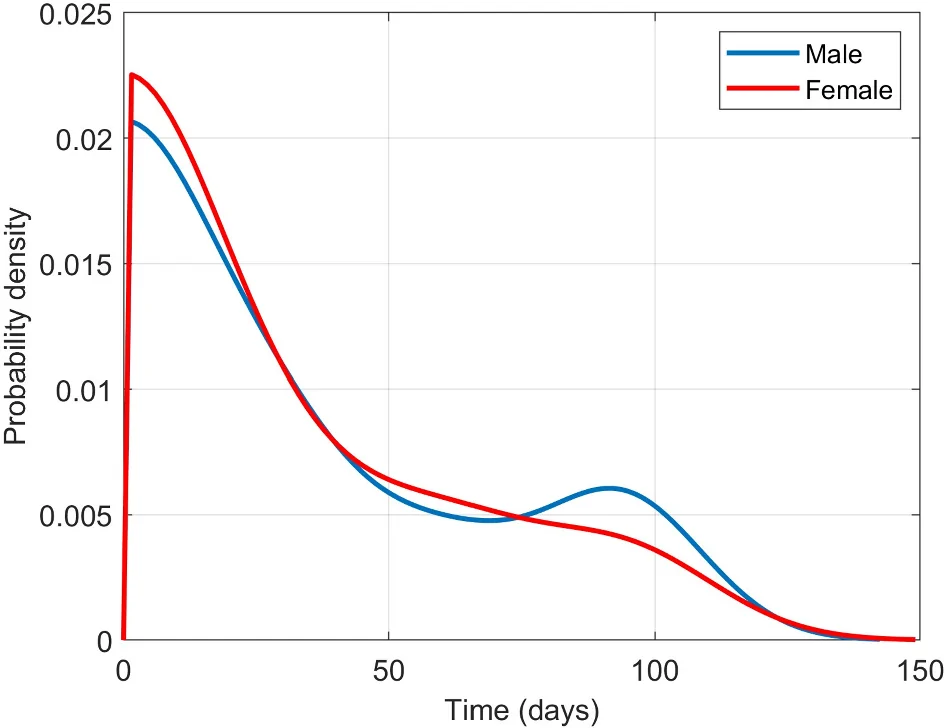
Tuberculosis diagnosis delay distributions for females and males from Kampala, Uganda. The distributions are kernel density estimates with standard normal kernel functions and positive support using data from Nsawotebba et al. (2025) (Color figure online)

The probability of transmission between a potential infector-infectee pair has been made partially dependent upon gender. Males are more likely to be infected than females, and transmission between individuals of the same gender is more likely than transmission between individuals of differing genders due to differences in daily contact rates resulting from increased sex assortativity of contacts (Miller et al. 2021). It is worth repeating in this section that an individual’s diagnosis delay when in the Active TB compartment is determined according to their gender, with males having a longer average delay than females: this may be due to differences in treatment seeking behaviour. The diagnosis delay distributions for females and males are shown in Fig. 2. In addition, the probabilities of transitioning from the Latent TB compartment to the Active TB compartment and from the Active TB compartment to the TB death compartment are scaled according to assigned sex (which we have assumed is aligned with gender identity, as noted above), to reflect the differences in disease progression rates; specifically the fact that males are more likely to become sick from, and die from, TB compared to females (Gao et al. 2017; Jimenez-Corona et al. 2006; Fox et al. 2016). Finally, it is worth repeating that we have allowed individuals with active TB to have cavitary disease: individuals with cavitation have a higher transmission rate (Urbanowski et al. 2020; Balogun et al. 2021). Evidence suggests males are more likely to develop cavitary tuberculosis (Balogun et al. 2021).

#### Place Size Distributions

**Fig. 3:**
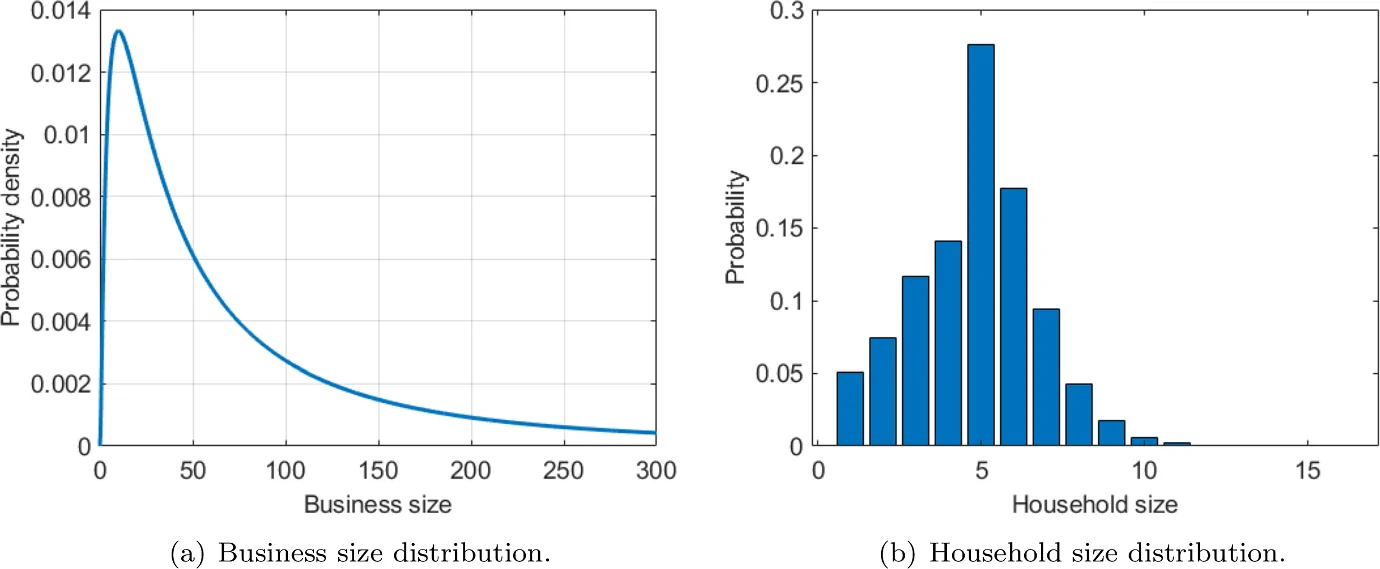
Assumed log-normal distribution of Kampalan business sizes (by number of employees), with mean and standard deviation taken from Goyette (2014), and assumed distribution of Kampalan household sizes, based on Uganda Bureau of Statistics (2024) and Jarosz (2021). Households of size 1 to 5 have probabilities taken from Uganda Bureau of Statistics (2024); households of size 6 or more are calculated based on the method given in Jarosz (2021)

We have changed Epiabm so that workplace sizes are drawn from log-normal distributions instead of power-law distributions, due to insufficient information on Ugandan business sizes to be able to fit them to a power-law distribution. We have based our assumption that Kampalan business sizes are log-normally distributed on Gibrat’s law of proportionate effect, which states that the growth rate of a business is independent of its current size (Samuels 1965). A brief justification of why business size should be log-normally distributed if Gibrat’s law holds is provided in Appendix D. Figure 3a shows the assumed probability distribution for Kampalan business sizes used in our model.

#### Additional Alterations

To increase the speed of the initialisation of the model, in households with three or more individuals, the mean child age gap parameter was removed so that each child is assigned an age between the minimum and maximum child ages without deliberately spacing their ages. The model initialisation has also been modified to allow a user-specified number of individuals to belong to the ‘Latent TB’, ‘Active TB’, ‘Under treatment’ and ‘Recovered but could relapse’ compartments. We have changed how the susceptibility of an individual is calculated by implementing a Who Acquired Infection From Whom (WAIFW) matrix to determine how susceptible an individual in one age group is to one from another. Additionally, we have added a WAIFW matrix stratified by gender, to account for research that suggests same-gender transmission appears to be more common than cross-gender transmission (Miller et al. 2021). We have altered how vaccination is implemented within the model, so that an initial proportion of all individuals is vaccinated and any newborn individuals are vaccinated with a certain probability thereafter. This is to reflect the fact that TB is an endemic disease in Uganda and most individuals are vaccinated by their first birthday, in contrast to COVID-19 which was a newly-emerging epidemic where the population had to be vaccinated during the initial outbreak with priority given to those more at risk.

### Parameter Estimation, Assumptions and Limitations

We have assumed that the transition times between the ‘Latent TB’ and ‘Active TB’ compartments (i.e. the latent period), between the ‘Active TB’ and ‘TB Death’ compartments (i.e. the infectious period), between the ‘Under treatment’ and ‘Active TB’ compartments (i.e. the time to stopping treatment early), and between the ‘Recovered but could relapse’ and ‘Active TB’ compartments (i.e. the time to relapse) are exponentially distributed. For transitions where we had data on the cumulative probabilities of transitioning between states after certain lengths of time (e.g. from Esmail et al. (2014); Borgdorff et al. (2011)), but not the mean length of time between transitions, we fit these distributions using the fminsearch function on MATLAB to find the parameter value that led to the best-fitting exponential distribution.

The assumptions that the infectious period and time to relapse were exponentially distributed were based on work by van den Driessche et al. (2007). The assumption that the latent period is exponentially distributed was also used in the model developed by Castillo-Chavez and Feng (1997). It was later shown that replacing this assumed distribution with arbitrary latent period distributions led to similar results (Feng et al. 2001); this justifies our model structure, which is simpler than some other epidemiological models of tuberculosis. To check this assumption was reasonable, we investigated a Gamma-distributed latent period with shape parameter 2. We found that, although there are fewer total case numbers in the first year compared to an exponentially-distributed latent period, total case numbers are almost identical by the end of simulations for both distributions and the male-to-female case ratios do not show much difference: for more details, see Appendix E. The assumption that the time to stop treatment is exponentially distributed is equivalent to that used in Prats et al. (2016), where individuals had a fixed daily probability of stopping treatment early, and whether they abandoned treatment or not was checked daily. Transition times between the ‘Under treatment’ and ‘Recovered but could relapse’ compartments (i.e. the treatment period) are assumed to be constant: individuals transfer between the two compartments at the moment the full treatment period has been completed.

The diagnosis delay distributions for each gender were both derived using kernel density estimation with Gaussian kernels and positive support (see Fig. 2). For households with six or more individuals (up to the maximum household size, stated in Table 5 in Appendix C), we have assumed the proportions of households comprising a given number of people are Poisson-distributed with the mean equal to the average household size minus 1, based on research by Jarosz (2021). That is, we start with a household occupied by one person, and the Poisson distribution determines the probability of a certain extra number of occupants living in that household. The entries in the WAIFW matrix are assumed to be proportional to contact rates between the age groups, based on research by Wallinga et al. (2006). We have assumed that all individuals are equally likely to transition between two given compartments regardless of age or gender. Estimates for the proportion of active TB cases with cavitation vary between 20% and 90% (Gadkowski and Stout 2008; Prats et al. 2016; Zhang et al. 2016; Urbanowski et al. 2020), so we have assumed a proportion of 40%. We have assumed that individuals with a cavitated form of the disease are 2.5 times as infectious, in keeping with the findings of a systematic review and meta-analysis of infectiousness risk factors for individuals with active TB (Melsew et al. 2018). Finally, we have assumed that the household and school/workplace attack rates are equivalent, as McIntosh et al. gave 95% uncertainty intervals for the ratio of the two quantities for different age groups in Uganda, all of which included 1 (specifically, (0.42, 8.47) for young children, (0.40, 5.12) for older children, and (0.20, 5.98) for adults) (McIntosh et al. 2019).

A full list of the numerical parameter values we used in our reference parameter set, and the justifications for using them, are given in Tables 4 to 12 in Appendix C. It is worth noting that not all parameter values could be determined using Kampala-specific (or Uganda-specific) data.

## Results

In this section, we present the outputs of the EpiabmTB model. Firstly, we confirm the model is able to replicate real-world data with calibrated parameter values. Next, we explore counterfactual scenarios to determine the relative impacts of certain physiological and behavioural factors in causing a high TB burden in males in Kampala, in order to deduce which have greater explanatory power in producing the differences observed between genders. In addition, we conducted a consistency analysis to determine how many simulations were required to ensure outputs like averages and uncertainty intervals were consistent. For more details on the consistency analysis, please see Appendix F. We also investigated the possibility of super-spreaders causing a majority of *M. tb* transmissions in our model: for more details, please see Appendix G.

### Calibrated Results

We applied a scale factor to the population of Kampala to reduce computational cost, and parametrised the model such that the initial simulated population structure matched the observed population structure for our calibrated results. We manually calibrated the model following the principles of CaliPro, a calibration method for fitting parameters to complex biological models (Joslyn et al. 2020). For more details regarding our choice of scale factor and the calibration process, please see Appendix H.

**Fig. 4:**
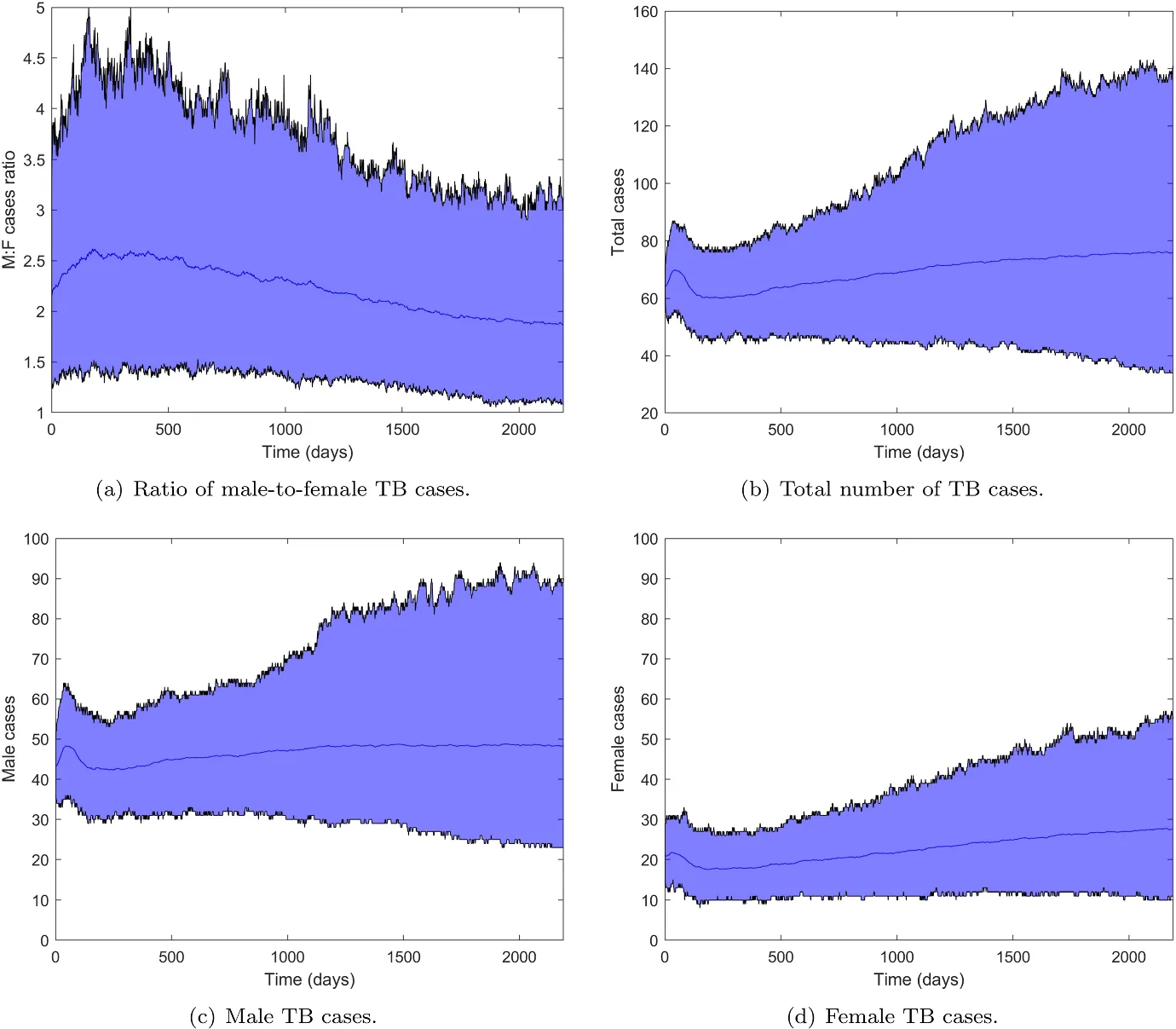
Summary plots of the male-to-female TB case ratio and total TB case numbers for all 300 simulations of the EpiabmTB model, as well as the TB cases stratified by gender. The shaded regions and the boundary lines indicate the 95% uncertainty intervals. The solid lines contained within these regions indicate the mean amounts of the random variable in question

After doing this, and subsequently completing the consistency analysis with the parameters found, we conducted 300 simulations of TB transmission in Kampala over a six-year time period, from 2017 to 2023. A summary of the 300 calibrated simulation results is shown in Fig. 4. As can be seen, the number of TB cases grows each year on average, rising from the initial condition of 65 total cases to approximately 76 on average. However, the 95% uncertainty intervals show that some simulations did see a decrease in the number of cases to fewer than 40 agents (34 was the lower bound of the uncertainty interval at the last time point of the simulations). Other simulations saw cases rise much more substantially over the time period simulated, with at least 140 total cases (the upper bound of the uncertainty interval for the last time point of the simulations was 140). This broadly matches the uncertainty in estimates of the real number of TB cases in Uganda over this time period: were the population of Uganda scaled down to be equal to the size of our simulated populations, the number of new TB cases per year from our simulations closely match the numbers that would be expected in reality (World Health Organization 2024a). The ratio of male-to-female TB cases is, on average, around 2 to 3 male cases for every female case for most of the simulated time period: the average ratio at the end of each simulation was 1.88 (to 2 decimal places). The 95% uncertainty intervals show that, in some simulations, the ratio almost drops to 1 male case for every female case (the lower bound of the uncertainty interval at the last time point of the simulations was 1.09 to 2 decimal places). In other simulations, it rises to between 3 and 5 male cases for every female case (the upper bound of the uncertainty interval for the last time point of the simulations was 3.13 to 2 decimal places). Again, this is consistent with what has been observed in Uganda in reality (Boum et al. 2014).^1^

### Counterfactual Scenarios

To determine to what extent certain differences between genders led to a higher TB burden in males, we compared the calibrated results to nine counterfactual scenarios. In Scenario 1, we symmetrically averaged all sex-specific and gender-specific parameter values for both genders (that is, we used the midpoint of the male and female parameter values) and removed polygamy. In Scenario 3, we symmetrically averaged any parameters involved in determining the within-host disease progression for both sexes (by using the midpoint of the male and female within-host parameter values) while leaving other parameters unchanged. In Scenario 5, we symmetrically averaged any behavioural parameters for both genders (by using the midpoint of the male and female place type percentages, WAIFW matrices, and initial case proportions, and fitting one kernel density estimate of the diagnosis delay distribution to all the available data that was then assigned to all individuals). In Scenario 7, we symmetrically averaged any parameters involved in generating diagnosis delays for both genders (by fitting one kernel density estimate to all the diagnosis delay data and assigning this distribution to all individuals) while leaving other parameters unchanged. Scenarios 2, 4, 6 and 8 are equivalent to Scenarios 1, 3, 5 and 7 in terms of the parameters changed, but are ‘directional’ counterfactuals: the male parameter values are set to the baseline female parameter values, while the female values are left unchanged from the baseline scenario. Finally, in Scenario 9, we removed polygamous marriages while leaving other parameters unchanged. A summary of which parameters were set equal (or set to zero in the case of the proportion of polygamous marriages) for each counterfactual can be found in Table 1. Summaries of the mean, median and standard deviation of the total case numbers and male-to-female case ratios at the last time point of each simulation for the baseline scenario and each counterfactual scenario are given in Tables 2 and 3.

**Table 1:** List of the parameters set equal (or set to zero in the case of the proportion of polygamous marriages) for each counterfactual scenario

Parameter	1	2	3	4	5	6	7	8	9
Male TB cases cavitated %	✓	✓	✓	✓					
Female TB cases cavitated %	✓	✓	✓	✓					
$\mathbb {P}$(male latent TB activation)	✓	✓	✓	✓					
$\mathbb {P}$(female latent TB activation)	✓	✓	✓	✓					
$\mathbb {P}$(male death via TB)	✓	✓	✓	✓					
$\mathbb {P}$(female death via TB)	✓	✓	✓	✓					
Male mean diagnosis delay	✓	✓			✓	✓	✓	✓	
Female mean diagnosis delay	✓	✓			✓	✓	✓	✓	
Male diagnosis delay ICDF	✓	✓			✓	✓	✓	✓	
Female diagnosis delay ICDF	✓	✓			✓	✓	✓	✓	
Males % by place type	✓	✓			✓	✓			
Females % by place type	✓	✓			✓	✓			
Case proportions by gender	✓	✓			✓	✓			
WAIFW matrix by gender	✓	✓			✓	✓			
Polygamy proportion	✓	✓							✓
Household partner age gap	✓	✓							

Scenario 1 (2 resp.) is all sex-specific and gender-specific parameters symmetrically averaged (set to female values resp.) for all individuals and polygamy removed; Scenario 3 (4 resp.) is within-host disease progression parameters symmetrically averaged (set to female values resp.) for all individuals; Scenario 5 (6 resp.) is behavioural parameters symmetrically averaged (set to female values resp.) for all individuals; Scenario 7 (8 resp.) is diagnosis delays symmetrically averaged (set to female values resp.) for all individuals; Scenario 9 is polygamy removed only. ICDF, inverse cumulative distribution function; WAIFW, Who Acquired Infection From Whom; resp., respectively

**Fig. 5:**
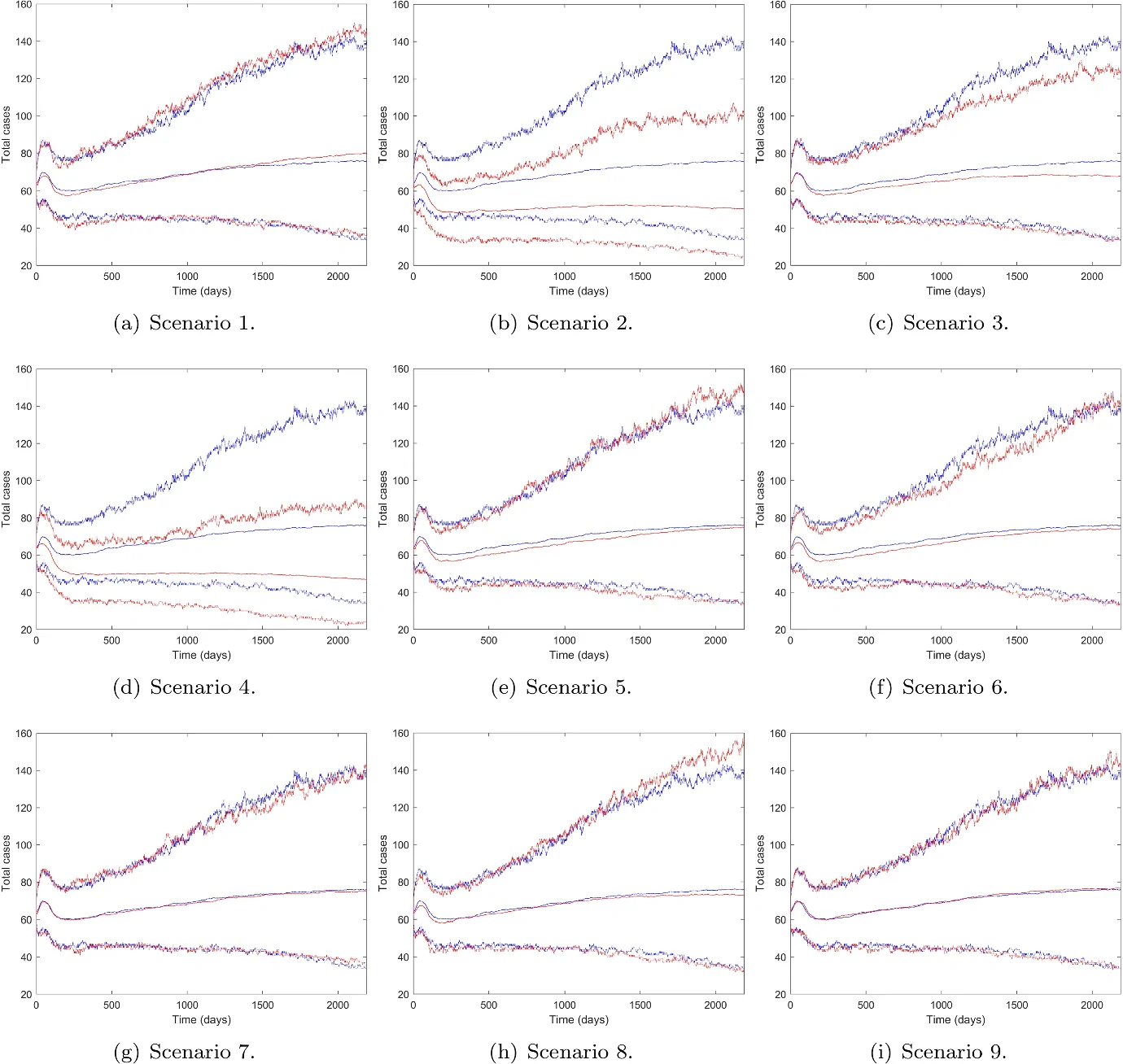
Summary plots of the total number of cases over 300 simulations of each counterfactual scenario, compared against the baseline calibrated scenario (i.e. the scenario with all gender differences included, presented in Sect. 3.1). The solid lines indicate the average outcomes, and the dashed lines indicate the 95% uncertainty intervals. The blue lines indicate the baseline calibrated scenario, and the red lines indicate the counterfactual scenario under investigation (Color figure online)

**Fig. 6:**
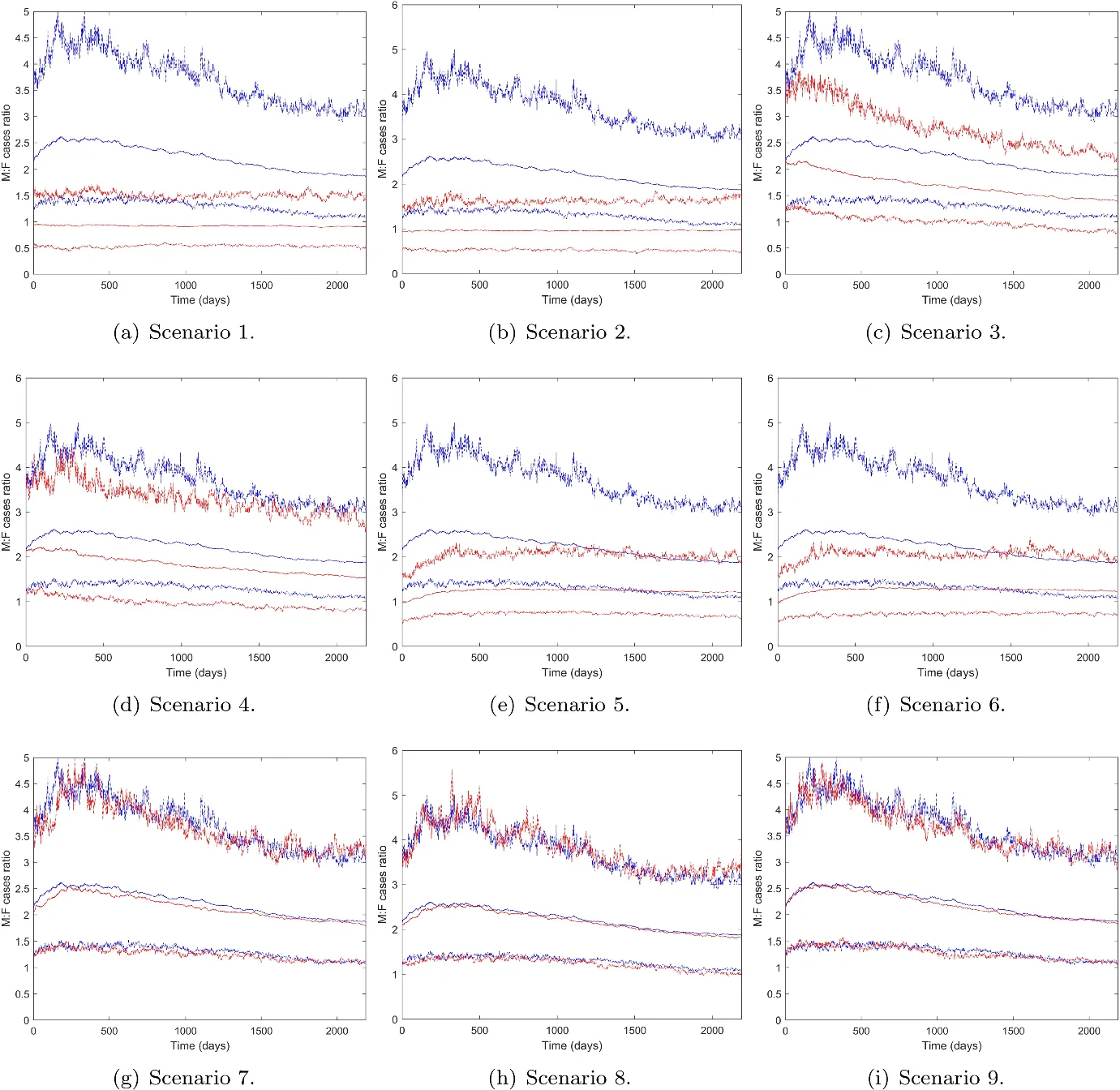
Summary plots of the male-to-female case ratio over 300 simulations of each counterfactual scenario, compared against the baseline calibrated scenario. The solid lines indicate the average outcomes, and the dashed lines indicate the 95% uncertainty intervals. The blue lines indicate the baseline calibrated scenario, and the red lines indicate the counterfactual scenario under investigation (Color figure online)

As can be seen from the plots in Figs. 5 and 6, some counterfactual scenarios led to substantial differences in the total number of cases and the male-to-female case ratio, whilst others were broadly similar to the calibrated results. As expected, in Scenarios 1 and 2, the male-to-female case ratio closely follows the male-to-female proportion across the whole population, which we had left as the calibrated parameter value. However, symmetrically averaging parameter values in Scenario 1 led to different results, in terms of total case numbers, when compared to setting all relevant parameters to the female values from the baseline scenario in Scenario 2. Scenario 1 led to a slight increase in the average number of cases: as the majority of cases were now in females, and we increased their diagnosis delay and probability of becoming sick when averaging these parameter values across both genders, their increase in cases was greater than the decrease in male cases that came from shorter male diagnosis delays and the lower probability of males becoming sick. On the other hand, in Scenario 2, all individuals have parameter values that either leave their risk of developing active TB identical or reduce it, so we see a reduction in total case numbers that is statistically significant at the 5% level of significance (see below for further details).

Scenarios 3 and 4 saw reductions in the average total number of cases by the end of each simulation that were statistically significant at the 5% level of significance (see below for further details), and the male-to-female case ratio moved closer to parity. In Scenario 3, sex differences in within-host parameters were removed by averaging, reducing male risk to the midpoint while simultaneously increasing female risk to the same midpoint. In Scenario 4, these differences were instead removed by setting the within-host parameters equal to the female values used in the baseline scenario for all individuals. This appears to have reduced the impact of the greater probability of transmission from males with active TB relative to females with active TB. Initially, it may seem counter-intuitive that Scenario 3 sees a greater change from the baseline scenario than Scenario 1, despite the fact that the parameters altered in Scenario 3 are a subset of the parameters changed in Scenario 1. This would imply that the additional parameters in Scenario 1 that were different in value to those used in the baseline scenario counteract the differences in the parameters changed in Scenario 3. We believe that this was the case: the fact that women (who made up a larger proportion of the population) had longer diagnosis delays, increased probabilities of being assigned workplaces/schools (thereby increasing transmission possibilities), increased probabilities of cavitation and more probability of an infectious contact with either gender in Scenario 1 than in the baseline scenario, combined with a larger proportion of initial cases, countered the reduction in the probability of transmission from males, present in both Scenarios 1 and 3. Scenarios 2 and 4 both led to reductions in total case numbers compared to the baseline that were statistically significant at the 5% level of significance (see below for further details), but unlike Scenarios 1 and 3, the differences between these two scenarios were smaller in magnitude.

Scenario 5 saw a decrease in the average total case numbers by the end of each simulation, although not by much more than in Scenario 7: the additional behavioural parameters beyond the diagnosis delay distributions did not have much effect in this regard. However, after starting at exactly 1, the male-to-female case ratio remains slightly above 1 on average for the rest of the simulation, without returning close to the baseline ratio. This would appear to provide support to the suggestion that high sex-assortative mixing of contacts contributes to the elevated male-to-female case ratio, a conclusion that is further supported by the similar results from Scenario 6.

In Scenario 7, there was a slight decrease in total case numbers and the male-to-female case ratio. The average male diagnosis delay was reduced by approximately 2.5 days, less than 10% of the previous value, so did not lead to a statistically significant reduction in the number of transmissions before starting treatment at the 5% level of significance (see below for further details); this would have also been somewhat offset by the slight increase in the average number of transmissions from females with active TB before they were diagnosed. The results for Scenario 8 are similar, with the reduction in total case numbers and the male-to-female case ratio not statistically significant when compared to the baseline scenario at the 5% level of significance (see below for further details).

Only a small proportion of households would have had polygamous marriages, so the small changes in total case numbers and male-to-female case ratio for Scenario 9 seem intuitive: removing this would likely only reduce household transmissions in what would have otherwise been polygamous households.

To check the statistical significance of the differences between the baseline scenario and each of the nine counterfactual scenarios, we conducted Mann–Whitney U tests comparing the endpoint distributions of the total case numbers and male-to-female case ratios. Summaries of the *p*-values are shown in Tables 2 and 3. In each case, the null hypothesis would be that the endpoint distribution from the baseline scenario and the endpoint distribution from the counterfactual scenario are samples drawn from continuous distributions with equal medians. We found we can reject the null hypothesis (that is, p<0.05) for the total case numbers in Scenarios 2, 3 and 4, and for the male-to-female case ratios in Scenarios 1 to 8. So the Mann–Whitney U tests would appear to suggest the total case numbers from the end time points of the baseline scenario are significantly different to those from Scenarios 2, 3 and 4, and that the male-to-female case ratios from the end time points of the baseline scenario are significantly different to those from Scenarios 1 to 8.

In summary, all counterfactual scenarios appeared to lead to a reduced male-to-female case ratio at the end of the six-year simulated time period on average, compared to the baseline calibrated results; other than in Scenario 9, these reductions are statistically significant at the 5% level of significance. Scenarios 1 and 5 to 9 did not lead to a significant change in the total number of cases compared to the baseline, whereas Scenarios 2, 3 and 4 led to a statistically significant decrease in total case numbers compared to the baseline.

**Table 2:** A comparison of the mean, median and standard deviation of the total number of cases at the end of each simulation between the baseline scenario and each counterfactual scenario considered (with scenario numbers given in brackets in the Scenario column), as well as the *p*-values generated from Mann–Whitney U tests comparing the endpoint distributions of the baseline scenario and the counterfactual scenario in question

Scenario	Mean	Median	Standard deviation	*p*-value
Baseline	76.09	69	28.68	
All sex-specific and gender-specific parameters symmetrically averaged (1)	79.78	74	31.37	0.19
All sex-specific and gender-specific parameters set to female values (2)	50.75	46	19.95	<0.001
Within-host parameters symmetrically averaged (3)	68.11	65	23.44	<0.001
Within-host parameters set to female values (4)	46.80	44	17.90	<0.001
Behavioural parameters symmetrically averaged (5)	74.51	69	30.16	0.29
Behavioural parameters set to female values (6)	74.13	68	27.82	0.42
Diagnosis delay parameters symmetrically averaged (7)	75.00	70	27.87	0.66
Diagnosis delay parameters set to female values (8)	73.04	66	29.74	0.08
Polygamy removed (9)	76.81	71	29.34	0.74

All values are rounded to 2 decimal places

**Table 3:** As for Table 2 but for male-to-female case ratios

Scenario	Mean	Median	Standard deviation	*p*-value
Baseline	1.88	1.80	0.55	
All sex-specific and gender-specific parameters symmetrically averaged (1)	0.91	0.88	0.23	<0.001
All sex-specific and gender-specific parameters set to female values (2)	0.98	0.93	0.30	<0.001
Within-host parameters symmetrically averaged (3)	1.40	1.34	0.37	<0.001
Within-host parameters set to female values (4)	1.55	1.43	0.50	<0.001
Behavioural parameters symmetrically averaged (5)	1.21	1.15	0.33	<0.001
Behavioural parameters set to female values (6)	1.22	1.20	0.30	<0.001
Diagnosis delay parameters symmetrically averaged (7)	1.79	1.69	0.54	0.02
Diagnosis delay parameters set to female values (8)	1.82	1.69	0.62	0.04
Polygamy removed (9)	1.83	1.75	0.57	0.30

## Discussion

We have been able to model tuberculosis transmission dynamics such that the output is consistent with what has been observed in the ongoing TB endemic in Kampala, by adapting the Epiabm framework first introduced by Gallagher et al. (2024). Specifically, by altering the disease states to be more specific to TB, by evolving the population’s socio-demography through ageing and reassignment to new households, workplaces and schools over time, and by stratifying the population by gender, we have generated case numbers and male-to-female TB case ratios that are consistent with the observed ranges of these quantities in reality. Furthermore, we found our model is capable of reproducing super-spreader events typically associated with TB transmission, as found in multiple previous studies (e.g. Ypma et al. (2013); McCreesh and White (2018); Melsew et al. (2019); Lee et al. (2020); Teicher (2023); Smith et al. (2023)) (please see Appendix G for more details). Finally, by considering nine counterfactual scenarios, we provided evidence for and against some of the possible factors that may lead to the higher TB case burden in males. In this way, our model provides a proof-of-concept framework that could inform policy after further validation.

The counterfactual scenarios where only the diagnosis delay was equalised (i.e. Scenarios 7 and 8) did not lead to a significant reduction in the total number of cases, but we set the length of diagnosis delay equal to the average across both genders in Scenario 7, and equal to the average female delay in Scenario 8; it would be interesting to consider best-case counterfactual scenarios, such as no diagnosis delays for any individuals with active TB, and how large a reduction in case numbers could be achieved as a result, as future work. The counterfactual scenarios also highlight the issue of sex assortativity in adult contacts. In Scenarios 3 and 4, the total number of cases and male-to-female case ratio were reduced when the within-host disease progression parameters were set equal, but the far greater likelihood of male-to-male transmission, compared to any other infector-infectee gender combination, meant the ratio did not approach parity. On the other hand, in Scenarios 5 and 6, equalising behavioural parameters, among them the entries of the gender-stratified Who Acquired Infection From Whom matrix for different combinations of infector gender and infectee gender, led to the male-to-female case ratio significantly decreasing compared to the baseline scenario. In Appendix G, we observed that super-spreaders can belong to either gender, and have both cavitary and non-cavitary TB. This emphasises the importance of rapid diagnosis for anyone with active TB, regardless of their individual characteristics. Having said this, we also found that super-spreaders are more likely to be male and are more likely to have cavitary TB; this suggests the diagnosis of individuals with either, or both, of these characteristics should be prioritised.

However, it should be acknowledged that our conclusions regarding the counterfactuals are conditional on a calibrated baseline generated with a single set of parameter values, rather than a range of acceptable parameter values - we have tuned a large set of free parameters to two endpoint targets to calibrate the model, from which all the counterfactual scenarios are run. As such, our parameter values may not be fully identifiable. It should also be noted that the within-host parameter values in the baseline scenario were all tuned, albeit with the ranges of potential values all taken from the literature, and thus Scenarios 3 and 4 are partially self-referential.

Furthermore, our calibration methodology could have been a fully automated approach. Our sampling of parameter values, pass/fail determination and refinement of the parameter space was conducted manually. An alternative approach would have been to follow the exact procedure outlined by CaliPro (Joslyn et al. 2020): conduct a Latin Hypercube sampling of the parameter space (or some alternative sampling technique); run simulations for each sampled parameter combination and perform pass/fail selection for each simulation; create two probability density distributions for each parameter - one for the ‘pass’ set and one for the ‘fail’ set - and narrow the parameter space using Highest Density Region or Alternative Density Subtraction selection; repeat the procedure until a pre-determined termination criteria has been reached (for example, a given percentage of simulations pass the evaluation process). Unlike this approach, we have calibrated the parameters to a single manually-tuned set of values, and the ‘pass/fail’ criteria for our calibration process - having the mean simulated outputs within the data-derived target intervals - was quite easy to satisfy, given the wide target uncertainty interval. Additionally, conducting a larger number of preliminary simulations would have provided greater confidence that our model was properly calibrated. Having said this, we believe that our model was calibrated sufficiently, in a systematic manner, with simulation outputs consistent with observed ranges for both total case numbers and male-to-female case ratios.

One potential limitation of the applicability of some of our counterfactual scenarios (specifically, Scenarios 1, 3, 5 and 7) is that our symmetric averaging approach effectively conflates two related but distinct questions: ‘What if males and females were biologically and/or behaviourally identical?’, and ‘What if males had the same biological and/or behavioural parameters as those observed in females in reality (or vice versa)?’ Our averaging approach answers the former question, but not the latter, which would arguably be more directly informative for public health by considering, for example, what would happen if the diagnosis delays for males were as short as the delays for females. The directional counterfactual scenarios (Scenarios 2, 4, 6 and 8) are much more helpful in this regard. Such directional counterfactuals could help support the Uganda National Tuberculosis and Leprosy Programme (NTLP) by evaluating programmatically relevant interventions, including targeted active case finding, workplace-based screening, alternative screening and case-finding strategies, and approaches to reduce diagnostic delays among men before implementation, thereby providing evidence to inform programme planning and prioritisation. As noted above, however, further validation is required before our model could be used to inform public health policy.

Our model has some further limitations. Perhaps the primary amongst these is that the agent-based model is computationally expensive. Consequently, it takes multiple hours to complete a single simulation when using a population the size of Kampala. As a result, we have had to reduce the size of the population and the period of time under investigation and we have had to use a supercomputer to complete all our simulations in a reasonable amount of time. Specifically, using the University of Bath’s Nimbus cloud supercomputer, each simulation of a six-year time period typically took around 50 min to complete with the scaled-down population. These simplifying assumptions may cause additional complications: our results may only be applicable to populations of a similar size over time frames of a similar duration.

Another limitation is that a number of parameter values have had to be estimated given the difficulty of observing the true values in the real-world data (the initial numbers of individuals in each compartment, for example). The counterfactual scenarios demonstrate the impact that altering certain key parameters can have on the results, but due to the complexity of the model, we have been unable to test the sensitivity of all model parameters, some of which may be having a substantial impact and could have feasibly taken alternative values. For example, the values chosen for the gender-stratified Who Acquired Infection From Whom matrix came from a single source, with the specific values chosen from the uncertainty intervals provided during the calibration process as they led to the best fit. Although we believed this source was reliable, other studies may suggest changing these estimates in future.

Additionally, as with other stochastic agent-based modelling frameworks, one needs to average a large number of simulations in order to be confident about the results; we have used the methodology proposed by Hamis, Stratiev, and Powathil to determine how many simulations are needed (Hamis et al. 2021), but a deterministic model would only require simulating once. Having said this, it is worth noting that deterministic modelling frameworks do not account for uncertainty in their results when provided with a given set of input variables and parameter values, something which our agent-based model is able to capture. Finally, it is worth noting that TB transmission is usually concentrated among key populations, for example, in areas with a high HIV prevalence, and among prisoners (Mathema et al. 2017). Therefore, the fact that our model does not take some of these key populations into account may have significantly confounded the gender analysis conducted, especially as Uganda has been identified by the World Health Organization as one of the 30 highest TB/HIV burden countries (World Health Organization 2025a). Given the high prevalence of HIV-associated TB in Uganda, the high male TB burden in Kampala may be partially explained by HIV co-infection. The heterogeneity in transmission between genders may have been better captured by altering our model to include these key populations, for example, by allowing for HIV co-infection, or including prisons as a place type.

### Future Work

With additional high-quality data, we could have added further features to our model. For example, we have assumed men in polygamous relationships have two wives, but if we had access to data that suggested otherwise (e.g. a probability distribution of the number of wives for Ugandan males), we could update the model accordingly. Having said this, Scenario 9 suggested polygamy has little impact on TB case numbers or the male-to-female case ratio, so it seems unlikely that increasing the number of wives in polygamous marriages in our model would change the lack of impact polygamy has on the output. It should be noted, however, that this finding depends on our conservative polygamy implementation.

Other features that could be implemented in future iterations of the model include age-specific natural death rates, immigration and emigration into and out of the city being modelled - Epiabm already has methods in place to deal with travellers in this regard - and different types of workplaces and spatial locations: there has been evidence that TB is more likely to be transmitted in certain places than others. For example, it has been shown that, in Dar es Salaam in Tanzania, TB is more likely to be spread in prisons and on public transport than in schools (Hella et al. 2017), so it would be interesting to see if this holds in other countries as well, especially as places with higher risk (e.g. prisons, public transport) are likely to have predominantly male workforces in some parts of the world. As mentioned in the previous section, we could add HIV co-infection as a property of individuals in the model to investigate as another possible cause of super-spreading.

Although we have used a model structure with progression from latent TB to active TB closely matching Structure E from the classification proposed by Menzies et al. (2018) (with the structures presented in more detail in Appendix B), better contact tracing data could potentially allow us to identify which of these structures best fits the data and to alter our model accordingly. Splitting the Latent TB compartment into two sub-compartments has been used in models similar to ours, such as the sex-stratified deterministic TB transmission model presented by Shaweno et al., with differential progression, reactivation and relapse rates (Shaweno et al. 2021). Compared to their approach, it is possible that our choice of model structure for latent infection and progression to active disease does not capture the high early activation risk and reduced later activation risk as successfully, although it does not appear to significantly change the total case numbers or male-to-female case ratio by the end of the six-year simulated time period (see Appendix E). It would be worth exploring this further in future work.

Finally, we could consider additional counterfactuals that focus on some of the between-host factors we did not investigate in this study. For example, by equalising just the proportions of males and females in schools/workplaces and the WAIFW matrix entries, we could determine the relative contribution of gender differences in mixing patterns. The counterfactual scenarios we did consider offer some indication of how sensitive the model output is to certain parameters, but a more thorough sensitivity analysis would reveal which parameters would be worth investigating further.

### Conclusion

In this paper, we have explored how sex- and gender-associated differences may influence TB transmission in Kampala. Specifically, through altering the Epiabm agent-based modelling framework, and using it to investigate the TB endemic over a six-year period, we have found that super-spreaders from both genders are likely to be the cause for the majority of transmissions - 20% of infectious individuals were found to cause approximately 80% of infections - and that within-host factors, such as the greater likelihood of cavitary TB and of progressing from latent to active TB, appear to substantially contribute to high total case numbers and a high male-to-female case ratio. In addition, the high sex assortative mixing of contacts also contributes to the elevated male-to-female case ratio, even when the within-host disease progression parameters are set equal. Our work therefore suggests both within-host and between-host factors influence the sex/gender disparity in TB case numbers, and should both be considered as reasons for the high male TB burden. Consequently, public health interventions looking to not only prevent cavitation, and the risk factors associated with it, but also to increase awareness of TB in men and the need for a reduction in social contacts whilst infectious (e.g. by self-isolating) are likely to prove impactful in reducing the severity of the TB epidemic in Kampala. Model outputs can inform targeted interventions such as increased male-focused screening.

## Figures and Tables

**Fig. 7:**
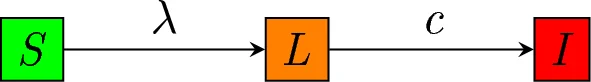
Structure A. Here, *S* is the susceptible compartment (labelled ‘Healthy’ in our model), *L* is the latent TB compartment, *I* is the active TB compartment, λ is the force of infection for *M. tb*, and *c* is the rate of progression from latent TB to active TB

**Fig. 8:**
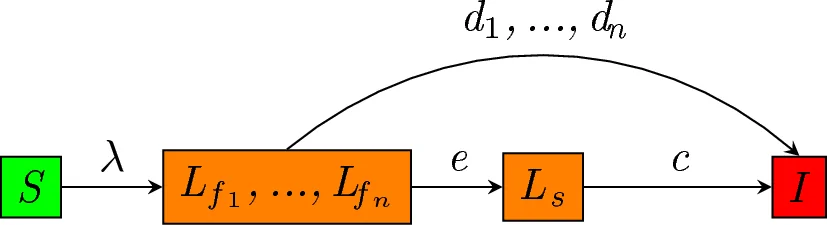
Structure B. Here, $L_s$ is the slow latent TB compartment, and $L_{f_1},...,L_{f_n}$ represent *n* ‘tunnel states’ of fast latent TB: if individuals in compartment $L_{f_i},i \in \{1,...,n-1\}$ do not transition to the active TB compartment in a given time step, they progress to state $L_{f_{i+1}}$. *e* is the rate of progression from fast latent TB to slow latent TB, and $d_1,...,d_n$ are *n* progression risks: individuals in compartment $L_{f_i},i \in \{1,...,n\}$ transition to the active TB compartment at rate $d_i$. All other symbols have the same meanings as in Fig. 7

**Fig. 9:**
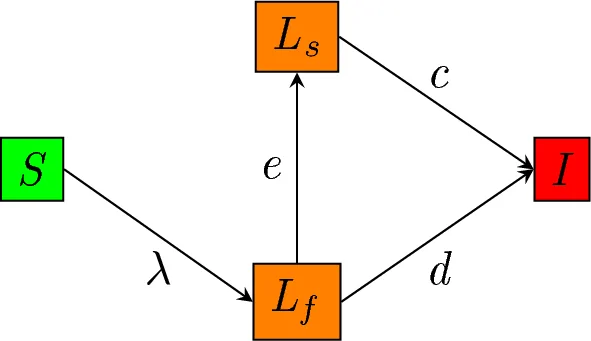
Structure C. Here, $L_f$ is the fast latent TB compartment and *d* is the rate of progression from fast latent TB to active TB. All other symbols have the same meanings as in Figs. 7 and 8

**Fig. 10:**
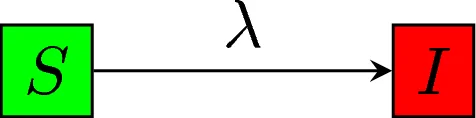
Structure D. All symbols have the same meanings as in Figs. 7, 8, and 9

**Fig. 11:**
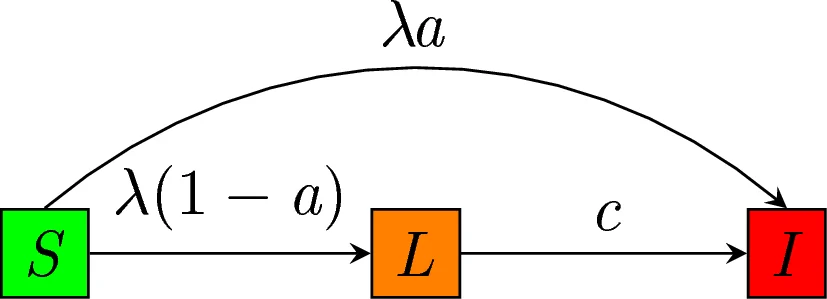
Structure E. Here, *a* is the probability of immediate progression from the susceptible compartment to the active TB compartment after *M. tb* infection. All other symbols have the same meanings as in Figs. 7, 8, 9, and 10

**Fig. 12:**
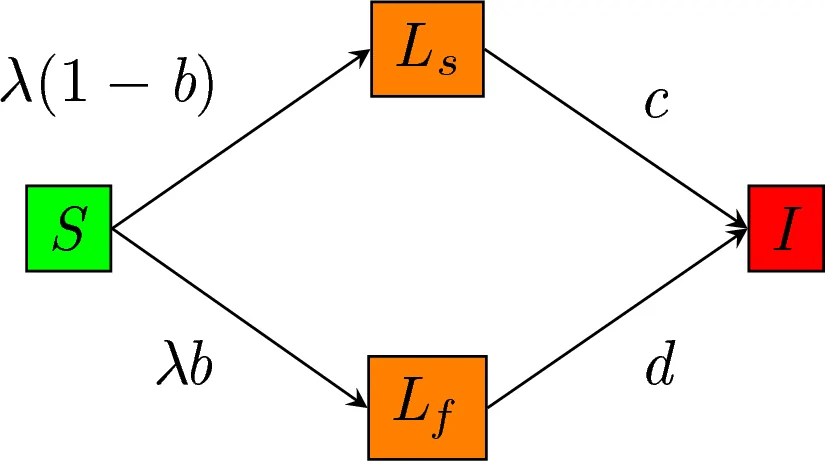
Structure F. Here, *b* is the probability of progression from the susceptible compartment to the fast latent TB compartment after *M. tb* infection. All other symbols have the same meanings as in Figs. 7, 8, 9, 10, and 11

**Fig. 13:**
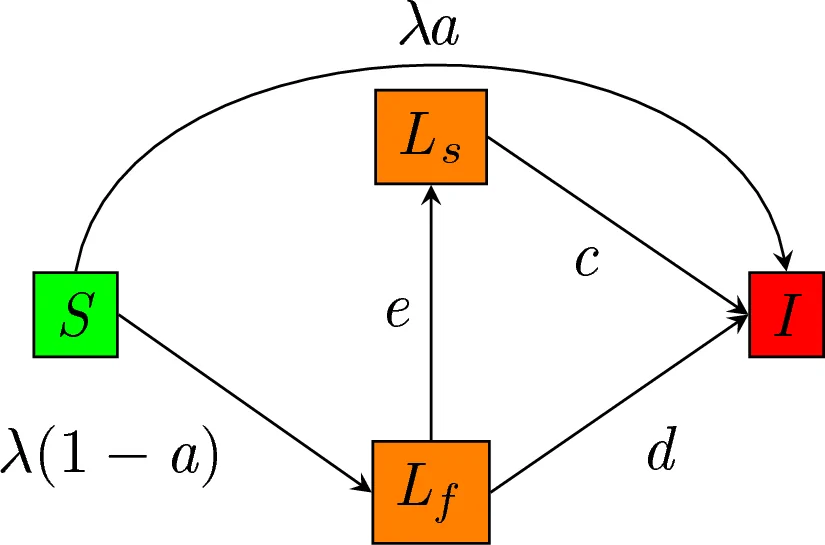
Structure G. All symbols have the same meanings as in Figs. 7, 8, 9, 10, 11, and 12

**Fig. 14:**
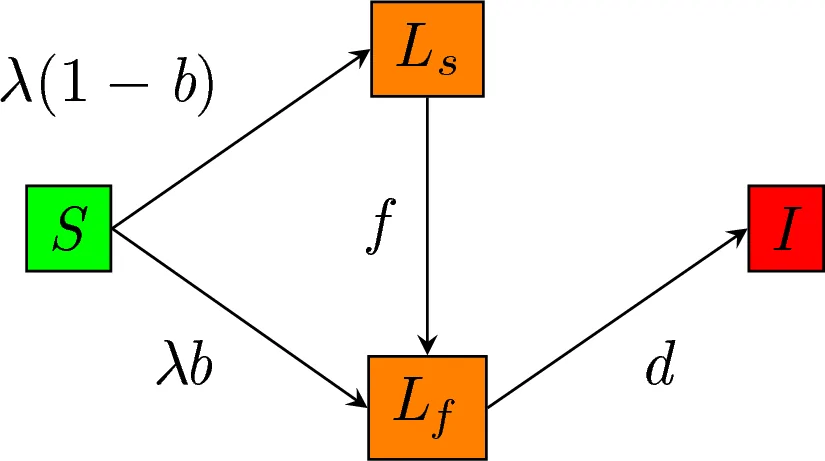
Structure H. Here, *f* is the rate of progression from the slow latent TB compartment to the fast latent TB compartment. All other symbols have the same meanings as in Figs. 7, 8, 9, 10, 11, 12, and 13

**Fig. 15:**
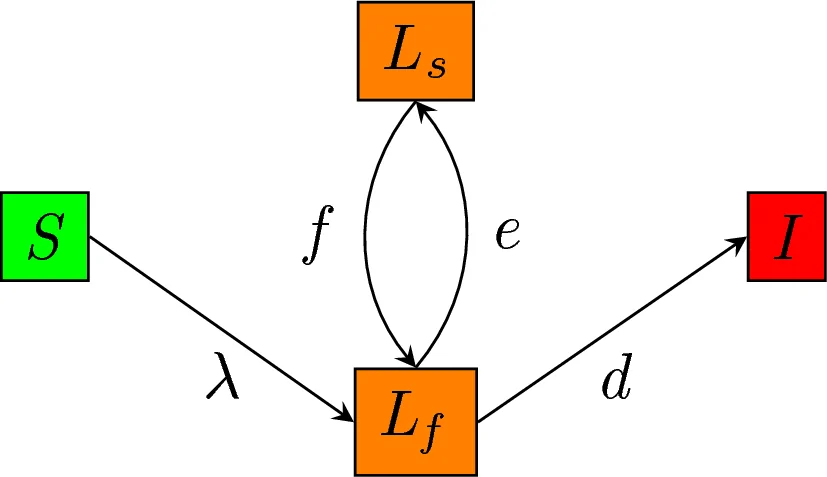
Structure I. All symbols have the same meanings as in Figs. 7, 8, 9, 10, 11, 12, 13, and 14

**Fig. 16:**
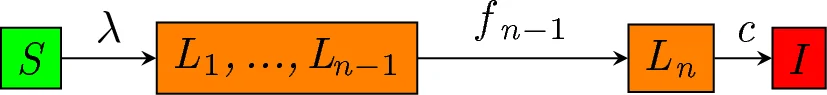
Structure J. Here, $L_1,...,L_n$ represent *n* sequential latent TB compartments: individuals in compartment $L_{i},i \in \{1,...,n-1\}$ transition to compartment $L_{i+1}$ at rate $f_i$. All other symbols have the same meanings as in Figs. 7, 8, 9, 10, 11, 12, 13, 14, and 15

**Fig. 17:**
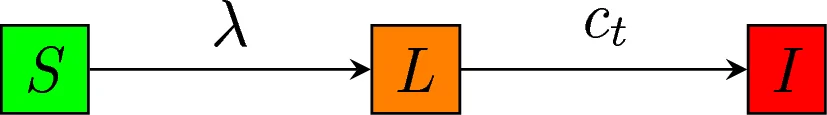
Structure K. Here, $c_t = x_1 \max (1,t)^{x_2}$ is the rate of progression from latent TB to active TB calculated as a function of an individual’s time since infection, *t*, and $x_1$ and $x_2$ are fitted parameter values. All other symbols have the same meanings as in Figs. 7, 8, 9, 10, 11, 12, 13, 14, 15, and 16

**Fig. 18:**
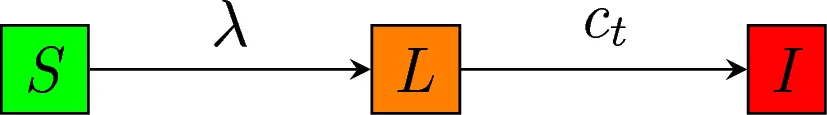
Structure L. Here, $c_t = x_1 (x_2 + \exp {(-x_3t)})$ is the rate of progression from latent TB to active TB calculated as a function of an individual’s time since infection, *t*, and $x_1$, $x_2$ and $x_3$ are fitted parameter values. All other symbols have the same meanings as in Figs. 7, 8, 9, 10, 11, 12, 13, 14, 15, 16, and 17

**Fig. 19:**
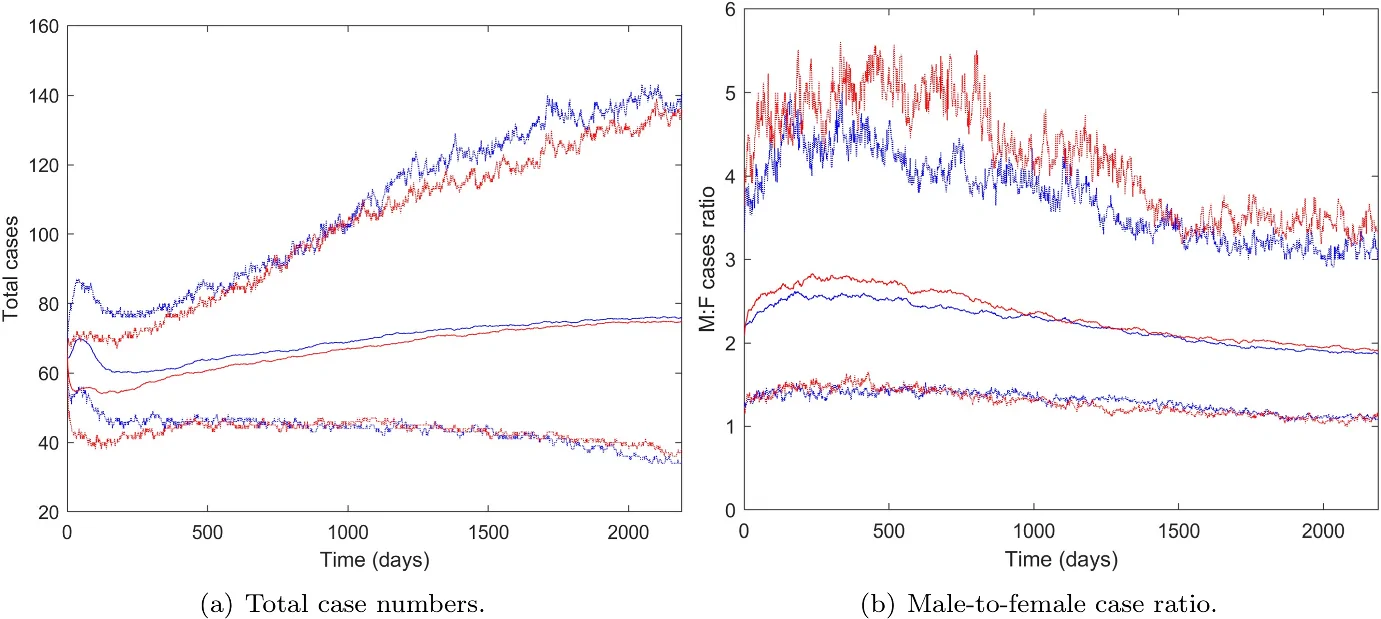
Summary plots of the total case number and male-to-female case ratio over 300 simulations with the latent period sampled from a Gamma distribution with shape 2, compared against the baseline scenario with an exponentially distributed latent period. The mean of the Gamma distribution is assumed to be equivalent to the mean of the exponential distribution. The solid lines indicate the average outcomes, and the dashed lines indicate the 95% uncertainty intervals. The blue lines indicate the baseline calibrated scenario, and the red lines indicate the counterfactual scenario under investigation, i.e. Gamma-distributed latent periods (Color figure online)

**Fig. 20:**
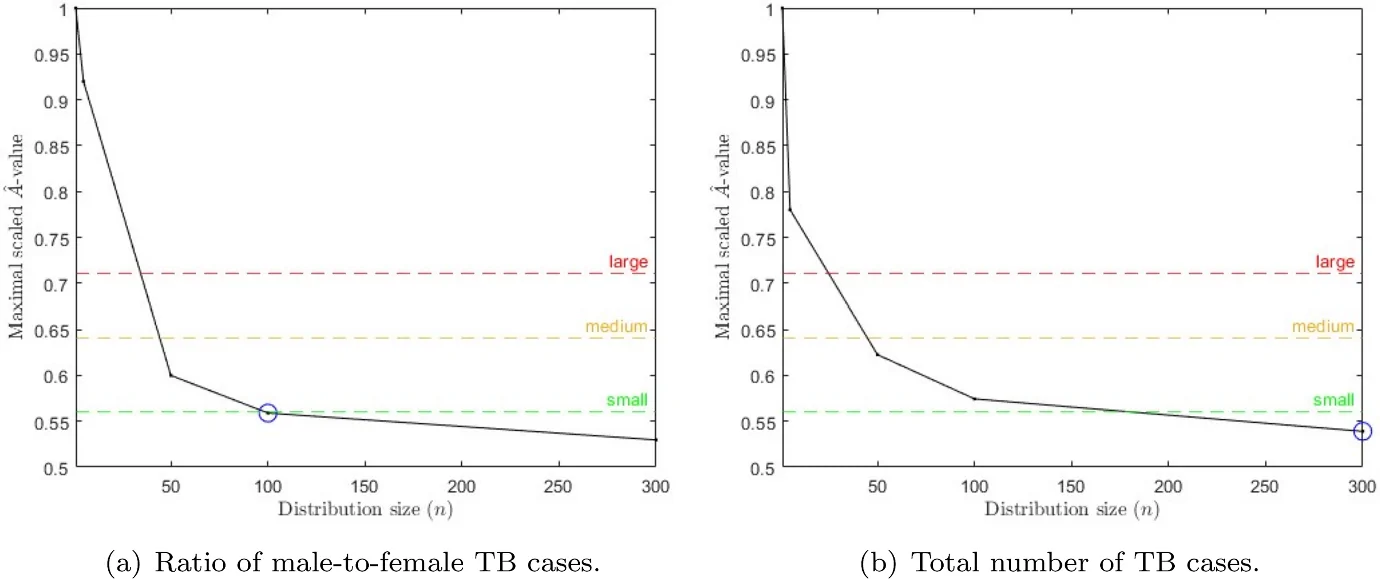
Consistency analysis for the EpiabmTB model. The maximal scaled $\hat{A}$-values for various distribution sizes *n* are shown. The red, yellow and green dashed lines indicate the thresholds below which the scaled $\hat{A}$-values have large, medium and small statistical significance in terms of the stochastic differences between samples of a given distribution size for the random variable being analysed. In the plot for each random variable analysed, the point surrounded by a blue circle is the $n^*$ value for that random variable, that is, the smallest value of *n* such that the maximal scaled $\hat{A}$-value for the random variable in question is below the threshold for small statistical significance in stochastic differences (Color figure online)

**Fig. 21:**
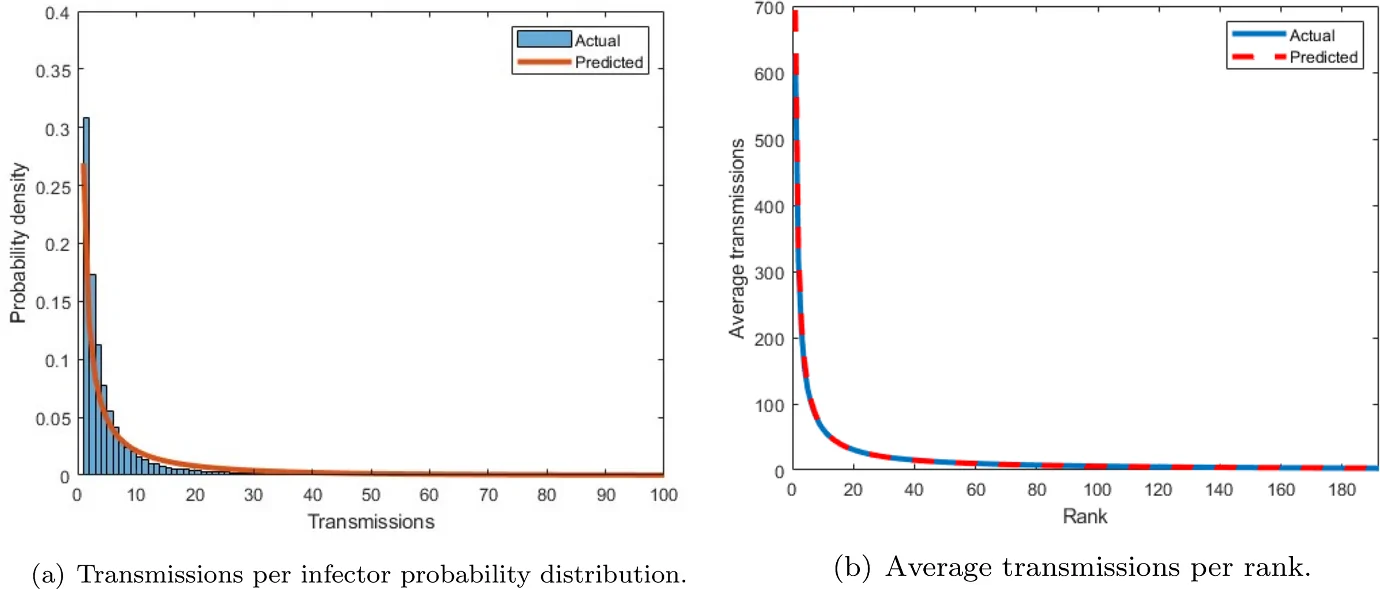
Probability distribution of the number of transmissions per infector (who infected at least one individual), averaged across the 300 simulations. The blue bars indicate the average probability density of the number of transmissions, and the orange curve shows the fitted zero-truncated negative binomial distribution; Average number of transmissions caused by the biggest transmitters in any given simulation across the 300 simulations, and the best-fitting power-law approximation. The blue solid line shows the actual average, and the red dashed line shows the predicted average, calculated using the power-law approximation (Color figure online)

**Fig. 22:**
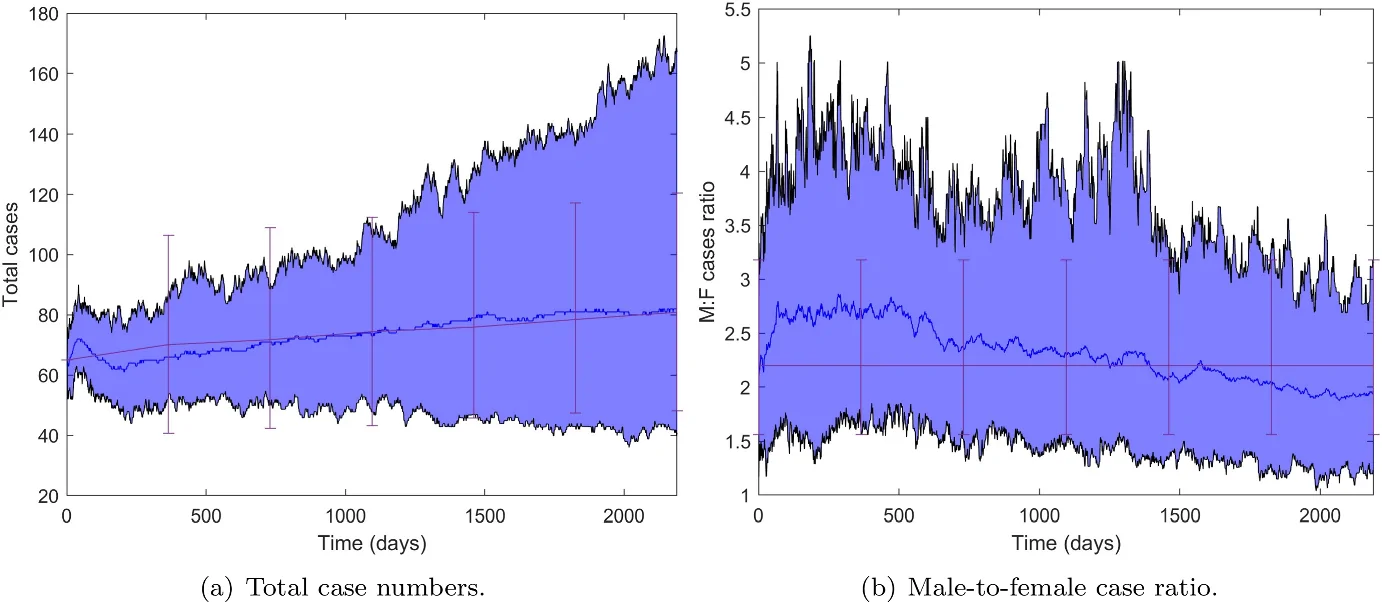
Summary plot of final 40 calibration runs, with the predicted 95% uncertainty intervals used as calibration targets (shown as error bars) and the actual 95% uncertainty intervals generated by the model (shown as the shaded blue regions). The solid blue lines within the shaded regions indicate the mean outcomes from the calibration runs. The predicted 95% uncertainty intervals for the year-on-year total case numbers came from the burden estimates of the World Health Organisation’s global tuberculosis report (World Health Organization 2024a), which were subsequently scaled to fit the predicted numbers of agents year-on-year in our model, based on a 2% scale factor being applied to the initial population size of Kampala and updated each year according to the rate of change of Kampala’s population size, which we have assumed is equal to the rate of change of Uganda’s population size (United Nations 2024c). The predicted 95% uncertainty interval for the male-to-female case ratio was taken from Boum et al. (2014) (Color figure online)

**Fig. 23:**
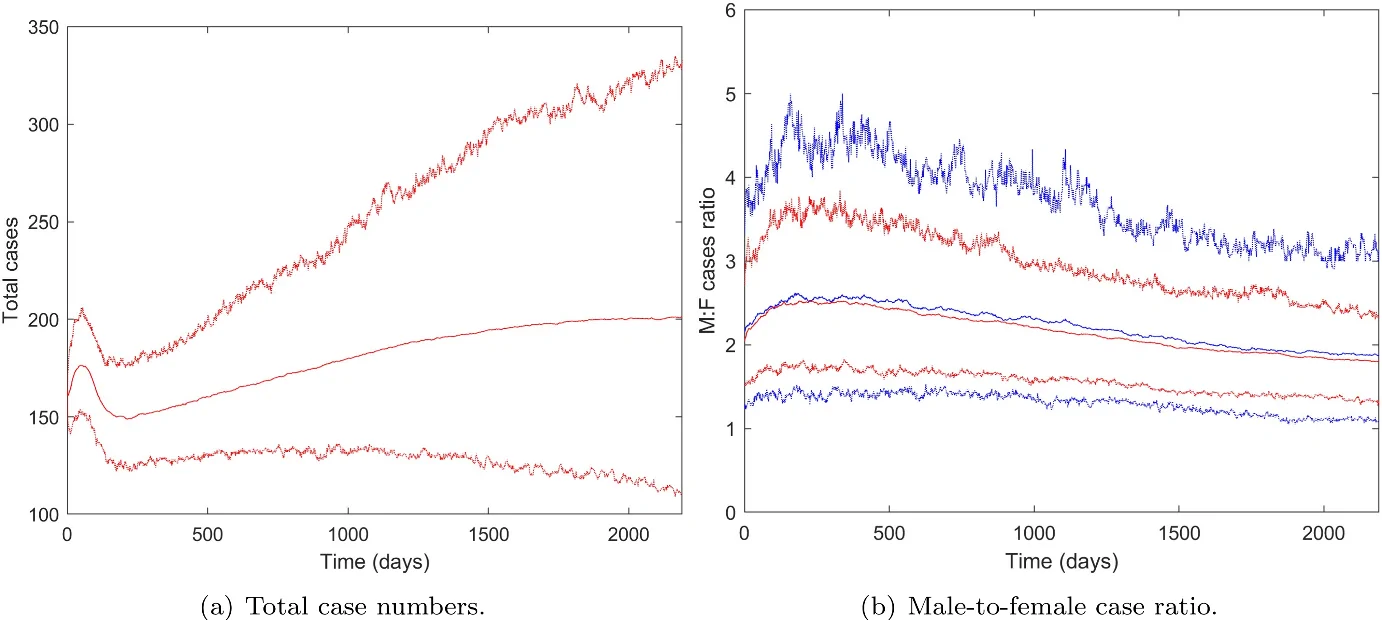
Summary plots of the total case number and male-to-female case ratio over 300 simulations with a 5% population scale factor applied. The solid lines indicate the average outcomes, and the dashed lines indicate the 95% uncertainty intervals. In the ratio summary plot, the blue lines indicate the baseline calibrated scenario with a 2% population scale factor applied; the red lines indicate the scenario with a 5% population scale factor applied (Color figure online)

**Fig. 24:**
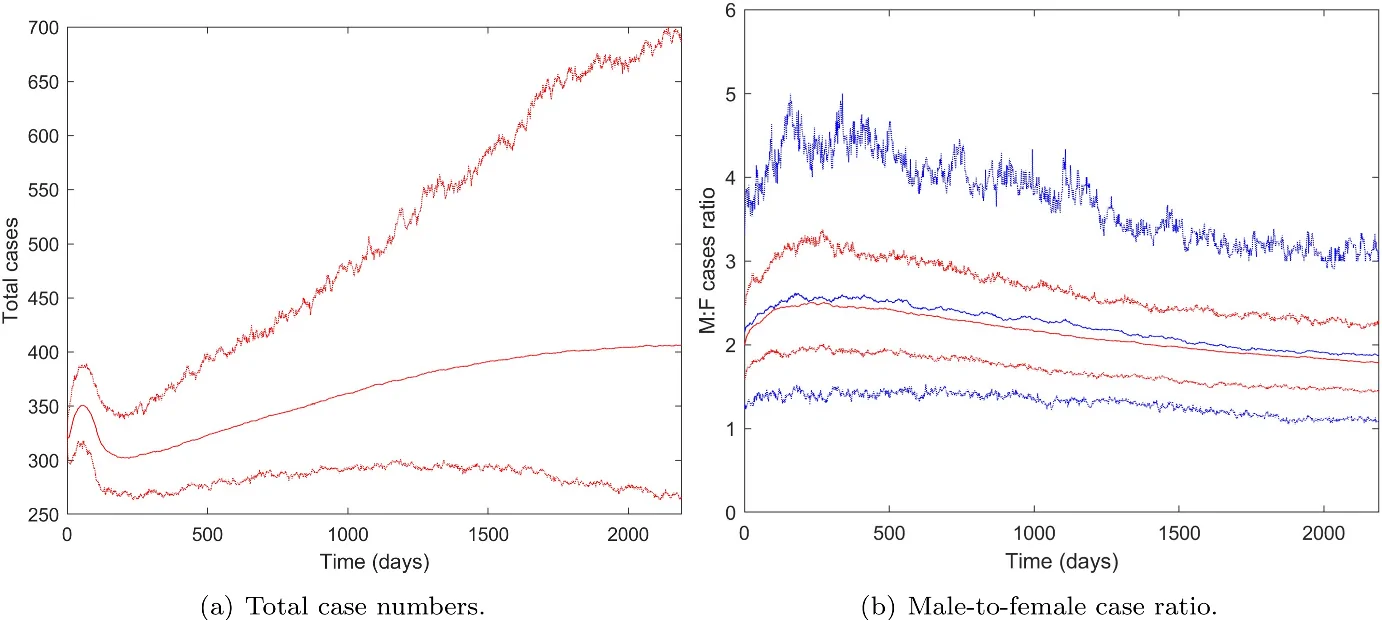
Summary plots of the total case number and male-to-female case ratio over 300 simulations with a 10% population scale factor applied. The solid lines indicate the average outcomes, and the dashed lines indicate the 95% uncertainty intervals. In the ratio summary plot, the blue lines indicate the baseline calibrated scenario with a 2% population scale factor applied; the red lines indicate the scenario with a 10% population scale factor applied (Color figure online)

**Fig. 25:**
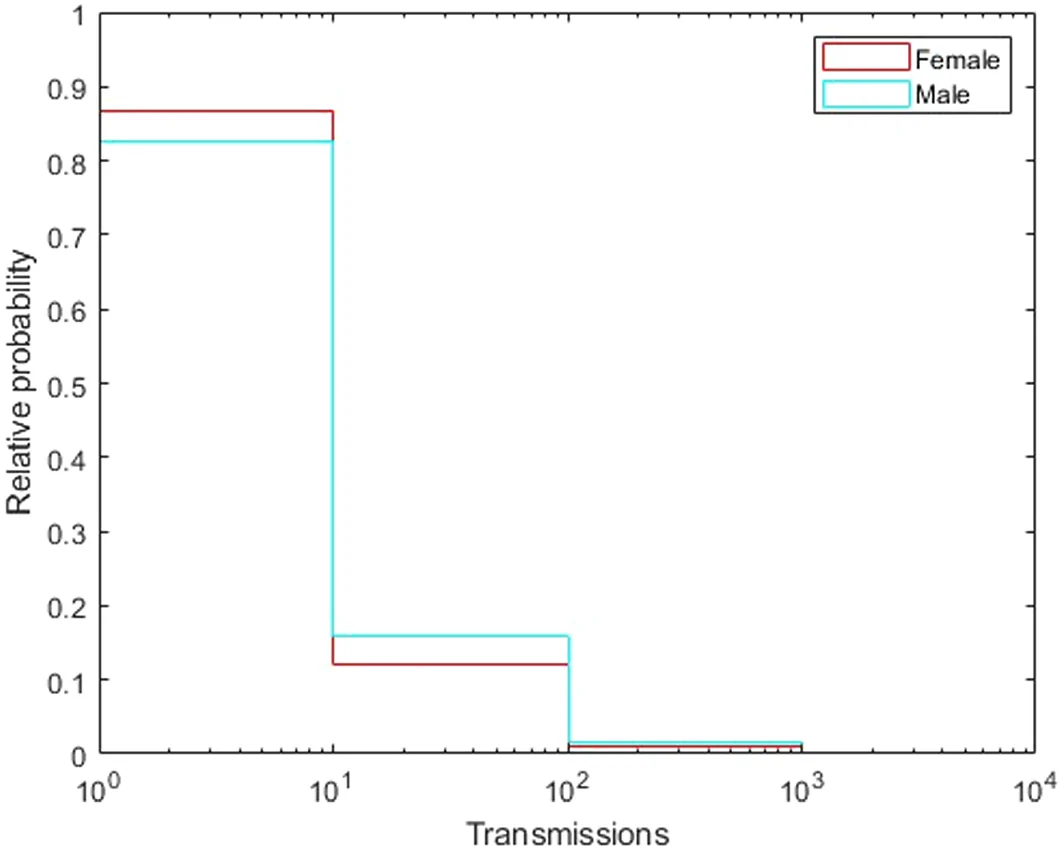
Probability distribution of the number of transmissions per infector (who infected at least one individual), stratified by gender, averaged across the 300 simulations. The red histogram bars indicate the average relative probability (that is, the number of elements in each bin relative to the total number of elements in the input data) of the number of transmissions per female infector, and the cyan histogram bars indicate the average relative probability of the number of transmissions per male infector. Note the *x*-axis is on a base-10 logarithmic scale (Color figure online)

**Fig. 26:**
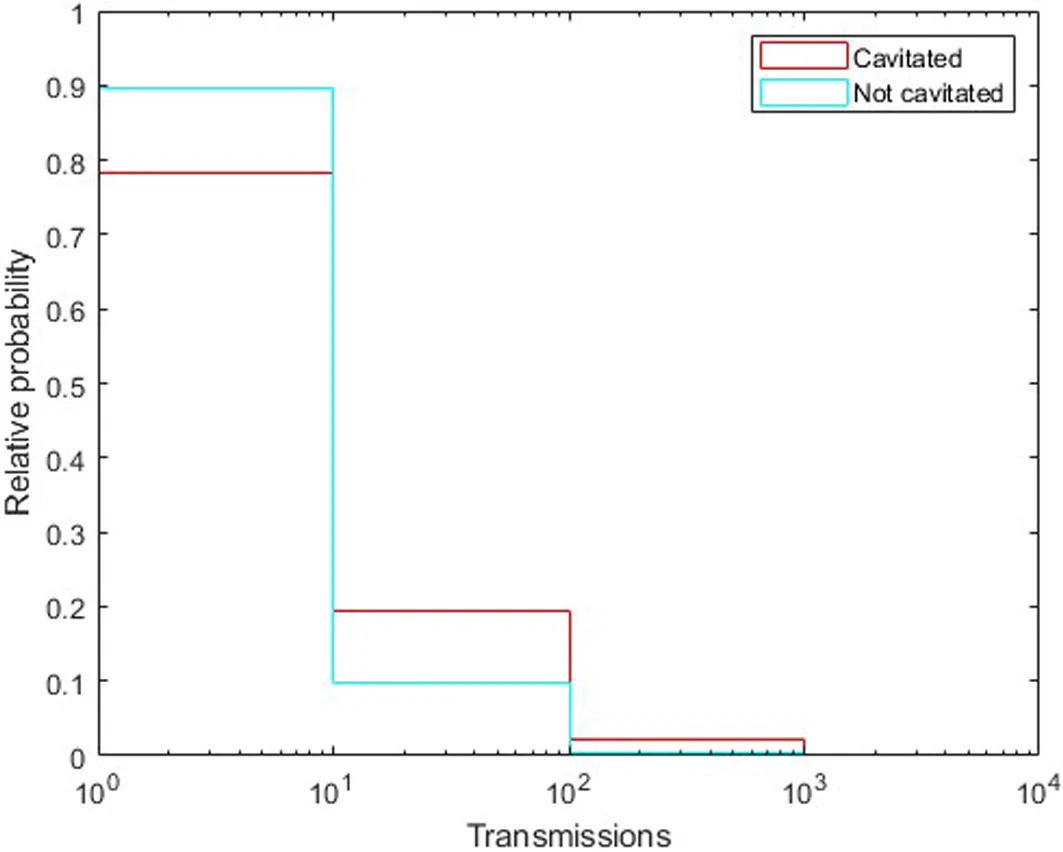
Probability distribution of the number of transmissions per infector (who infected at least one individual), stratified by cavitation status, averaged across the 300 simulations. The red histogram bars indicate the average relative probability (that is, the number of elements in each bin relative to the total number of elements in the input data) of the number of transmissions per infector with cavitary TB, and the cyan histogram bars indicate the average relative probability of the number of transmissions per infector with non-cavitary TB. Note the *x*-axis is on a base-10 logarithmic scale (Color figure online)

**Fig. 27:**
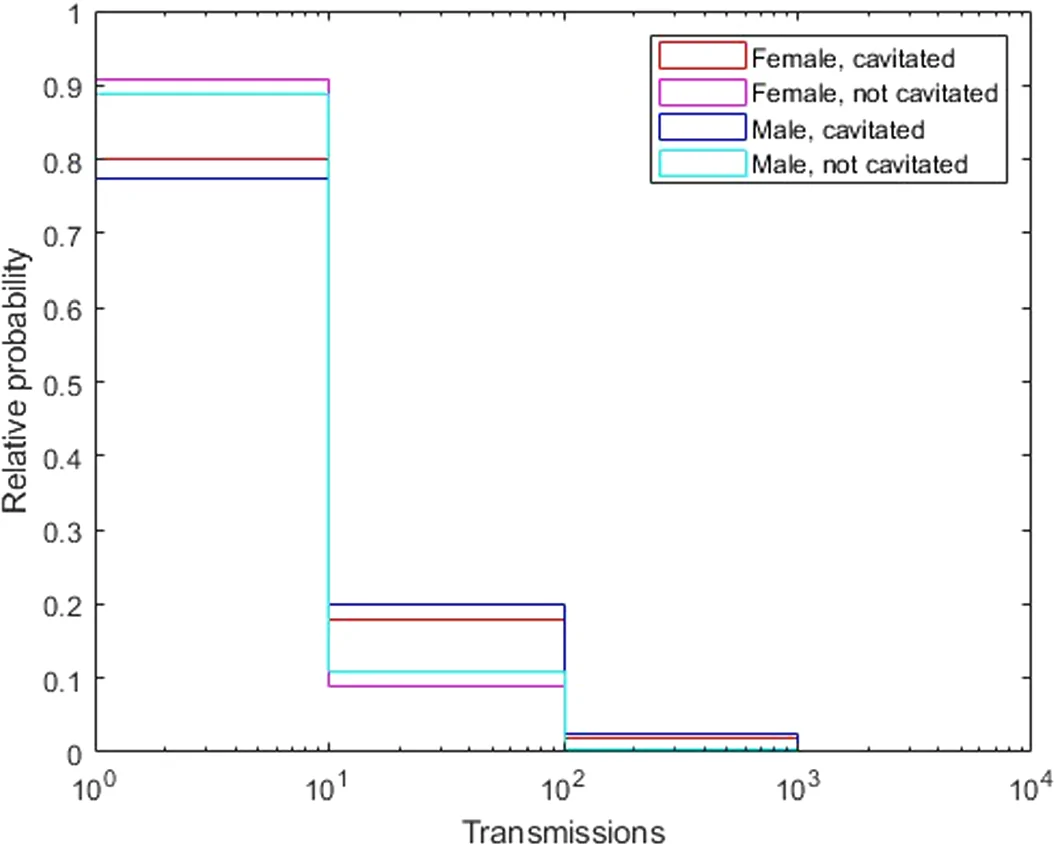
Probability distribution of the number of transmissions per infector (who infected at least one individual), stratified by both gender and cavitation status, averaged across the 300 simulations. The red histogram bars indicate the average relative probability (that is, the number of elements in each bin relative to the total number of elements in the input data) of the number of transmissions per female infector with cavitary TB, the magenta histogram bars indicate the average relative probability of the number of transmissions per female infector with non-cavitary TB, the blue histogram bars indicate the average relative probability of the number of transmissions per male infector with cavitary TB, and the cyan histogram bars indicate the average relative probability of the number of transmissions per male infector with non-cavitary TB. Note the *x*-axis is on a base-10 logarithmic scale (Color figure online)

## Data Availability

The model code can be found at https://github.com/jwgd93/EpiabmTB. The data presented in this article can be found at the following links: https://doi.org/10.5281/zenodo.18098364 for the consistency analysis data; https://doi.org/10.5281/zenodo.18098766 for the calibrated results data; https://doi.org/10.5281/zenodo.18098747 for the data from counterfactual scenarios 1, 3, 7 and 9; https://doi.org/10.5281/zenodo.20451803 for the data from counterfactual scenario 5 and Appendices E and I; https://doi.org/10.5281/zenodo.21500492 for the data from counterfactual scenarios 2, 4, 6 and 8; https://doi.org/10.5281/zenodo.21500554 for the calibration data.

## Appendix A CovidSim and Epiabm

### A.1 Overview

CovidSim is a spatial agent-based model, written in C++, in which individuals exist on a grid of square ‘cells’; each of these cells is further divided into ‘microcells’, with each microcell representing a small part of the geographic region being modelled. The model is set up so that each cell is split into 9 × 9 microcells, and the width of each microcell corresponds to 1/120 of a degree of latitude. Individuals are allocated to households and ‘places’ (that is, schools and workplaces) within these cells, based upon population density data for the region under investigation, and are assigned attributes, such as age and a corresponding 5-year age group (e.g. if an individual is 3 years old, they belong to the 0-4 age group). Individuals also have a current infectious disease state, which corresponds to those typically used in compartmental models of infectious diseases (i.e. Susceptible, Exposed, Infectious, Recovered and Dead/Removed). The infectious compartment is further sub-divided into the following categories: asymptomatic; mild; requiring a GP visit; requiring a hospital visit; requiring an ICU visit; recovering in the ICU but still infectious. This is to account for the fact that an infection from COVID-19 (which the model was originally developed to investigate) can vary in severity from person to person and that not all infectious individuals are symptomatic. The place to which an individual is assigned may be in a different cell to their household. Within each place, individuals are split into ‘place groups’, with individuals in the same place group being more likely to interact with each other than individuals in different place groups. They can interact with other individuals within their household, within their place, and via random social meetings which occur with a probability that decreases with distance from their household. It should be noted that the act of people moving is not explicitly modelled; the simulation uses spatial kernels to determine whether an infectious individual in one cell infects a susceptible individual in another cell. The model is initialised with most individuals belonging to the Susceptible compartment and the remainder being Infected. Infections can be seeded in a number of ways (see Sect. A.2 for more details). To determine where the infection spreads, a force of infection is calculated for each susceptible individual based on their interactions with infectious individuals.

Epiabm re-implements CovidSim as a more modular version of the original agent-based model and is fully documented; two backends are provided, one in Python and the other in C++. The code is written so that population generation and storage is more object-oriented than it is in CovidSim; although less efficient, the form of the code is more easily adaptable (Gallagher et al. 2024). Further details of how both models work are provided in the following sections.

### A.2 Initialisation of the Model

Populations in CovidSim and Epiabm are generated in one of two ways. If a population density file has been specified, both models read this and add people based on the information within the file. From this, individuals can be placed so that the number of people generated matches the expected number listed in the file for each cell and microcell. It is worth mentioning that CovidSim was released with pre-generated configuration files for some regions and countries, while Epiabm uses a separate software package, EpiGeoPop, to generate these files (Herriott et al. 2025). If no such population file is given, the population is generated in random locations based on the number of cells, number of microcells and total population size; this can be subject to constraints such as how many households and how many places there are in each microcell.

In both CovidSim and Epiabm, individuals are assigned ages based upon the number of people within their household, using a heuristic algorithm. The sizes of the households are determined before ages are assigned, using a household size distribution which is specified by the user within the parameter file. The algorithm can be summarised as follows:

- Determine the type of household given the household size, e.g. if the household consists of two people, the household could contain two younger adults, two older adults, or a single parent and their child, and determine the number of children within the household, if any are present;
- If there are children within the household:
  - Determine the age of the youngest child, subject to appropriate constraints;
  - Set the age of each subsequent child (if any) to be a Poisson-distributed number larger than the previous child’s age, subject to appropriate constraints, with the mean equal to the mean child age gap;
  - Choose the ages of their parents, subject to appropriate constraints;
- If there are children and additional adults beyond the parents within the household, assume they are grandparents and assign them ages such that the age gap between them and the parents is realistic.

CovidSim was built with the option to choose place sizes from either a Poisson, log-normal or power-law distribution. Workplaces and schools are generated and distributed in space according to population density files. In the case of schools, children ‘pick’ one of the nearest schools to them, with a probability that decreases with distance to the school. In the case of workplaces, workers ‘compete’ for vacancies with a probability that decreases with distance. These allocations are weighted with the probability of a person with a given age being in a certain type of place.

Epiabm removed the option to choose place sizes from log-normal distributions, instead choosing school sizes from Poisson distributions and workplace sizes from power-law distributions. It should be noted that these are the same distributions typically used for these place types in CovidSim. Otherwise, the two models are similar in this regard.

The user specifies the number of initial infections in both models. CovidSim allows the locations of the initial infections to be chosen in a number of different ways:

- The probability of initial infections being within a given microcell can be made to be dependent upon population density data, or;
- Certain locations can be specified to be the locations of the initial infections, or;
- The locations of the initial infections can be chosen at random (that is, from an initial infection location, find the next seeding location by determining the *x*- and *y*-coordinates using a radius and angle generated uniformly; set this subsequent location to be the new initial location and repeat until enough infections have been seeded).

The initialisation of infections in Epiabm is similar: locations for the initial infections can be specified by the user, otherwise the infections are seeded randomly throughout the spatial domain (that is, the model draws a random sample uniformly from all the people in the population to be the initially infected individuals).

### A.3 Running the Model

In Epiabm, once the model has been initialised, the code checks each member of the population one-by-one per time step using multiple functions (one for potential household infections, another for potential infections in places and one for potential transmission between individuals in different cells) to determine which infection events take place and where they occur. For individuals newly infected, they move to the Infected state and the subsequent progression of their infection is randomly decided according to the probabilities of moving from one infection state to another, and the time it takes for this change of status. Additionally, a further function updates the places of the individuals. If non-pharmaceutical interventions have been included, a function determines if any given intervention should be active given the time the policy has been in place and number of infected individuals. If travellers (i.e. individuals not permanently assigned to any specific place) enter or leave the location being modelled, another function determines when they enter the location, which household they reside in during their stay, and how long they stay, for each of those individuals. It is assumed they are infectious throughout their stay. As our model does not make use of non-pharmaceutical interventions and does not introduce or remove travellers, extra details regarding the functions controlling these aspects of the model are not included in this paper; more information can be found in Herriott et al. (2025). The mechanisms for updating the population and determining infection events and disease progression in the CovidSim model are similar to the Epiabm model unless otherwise stated.

Infection events can occur within households, within places, or outside of households and places (i.e. transmission between individuals in different cells). Separate ‘sweep’ functions for each of these possibilities determine how many infection events take place. In the case of household infection events, the susceptible people (‘infectees’) in the households of every infectious individual (the ‘infectors’) are each checked individually. In the case of place infection events, the infectees are members of the infector’s place group (e.g. classroom, office, etc). An alternative method is used to determine whether transmission between individuals in different cells occurs: for each cell, the number of infectious individuals is found, and their cumulative infectiousness is used to find a Poisson-distributed number of infections that the infectors in that cell will cause. A list of infectees from the cells other than the ‘infector cell’ is then determined by their distance to a randomly chosen infector. CovidSim and Epiabm differ in how distance affects whether somebody is a potential infectee in an infection event between individuals in different cells. Epiabm uses a cut-off distance, beyond which individuals cannot be infected by an infector in another cell; CovidSim calculates the ratio between the spatial kernel of the distance between the potential infector and infectee, and the spatial kernel of the minimum distance between their cells, adding the infectee to the list of potential transmission events if this ratio is greater than a number chosen uniformly at random between 0 and 1. The weighting given by the spatial kernel for distance *d* is given by the formula

1 $$\begin{aligned} \frac{1}{(1 + \frac{{\text {d}}}{{\text {a}}})^{\text {b}}}, \end{aligned}$$

where the scale parameter, *a*, and shape parameter, *b*, can be specified by the user. In addition to the cut-off method, this option is also available in Epiabm, where the scale and shape parameters have default values of a=b=1.

For each of these types of potential transmissions, a force of infection is calculated, and is compared to a number chosen uniformly at random between 0 and 1. This force of infection is based upon the infector’s infectiousness and the infectee’s susceptibility in that time step and will typically be a number between 0 and 1, corresponding to certain infection. These are each in turn impacted by whether any non-pharmaceutical interventions are in place and whether or not the infector and/or infectee are care home residents. If the number chosen uniformly at random between 0 and 1 is less than the force of infection, the transmission event occurs and the infectee is exposed to the disease, entering a latent state (the infection state is labelled as ‘Exposed’ in Epiabm). For place infection events, if the infector’s infectiousness is sufficiently large (that is, at least equal to 1), all infectees become infected. All infected individuals are assigned the Exposed infection state.

The within-host sweep checks each member of the population in turn. If the time their infection status was due to change (their ‘time of status change’) has been passed, the function updates their infection status and, if their new status is an infectious one, sets their infectiousness. The individual’s next infection status, and the time at which they will transition to this status, is then determined. Next, infection statuses and times of status changes are drawn from user-specified cumulative distribution functions. If the person’s time of status change has not been passed, and they are in an infectious state, their infectiousness is updated. Infectiousness is based upon a user-specified infectiousness profile that varies over the course of infection.

The places of individuals are updated at every time step. Each place is individually checked and the individuals associated with the place are updated. Whether an individual is added to, or removed from, a place will depend on the mean capacity of such places, the average size of place groups within such places (e.g. classroom sizes in schools), the probability distribution governing their size, and the probability of seeing a person of a certain age in that type of place (Fig. 18).

## Appendix B Model Structures for Progression from Latent TB to Active TB

The following model structures were identified by Menzies et al. (2018).

## Appendix C Parameter Values

See Table 4, 5, 6, 7, 8, 9, 10, 11 and 12 .

**Table 4:** Reference parameter set for initialising the model

Parameter description	Value	Justification
Population size	33,612	(Uganda Bureau of Statistics 2019), see §3.1
Number of cells	9	assumed
Number of microcells per cell	81	assumed
Number of households per microcell	11	(Uganda Bureau of Statistics 2024), see §3.1
Number of places per microcell	0.17	(Goyette 2014; Ministry of Education & Sports 2017), see §3.1
Simulation start time (days)	0	default
Simulation end time (days)	2,190	heuristically chosen
Initial number of people with latent TB	1,655	(Schwalb et al. 2026), calibrated, see §3.1
Initial number of people with active TB	65	(Aceng et al. 2024), see §3.1
Initial number of people receiving treatment	90	calibrated, see §3.1
Initial number of people that could relapse	425	calibrated, see §3.1

Units are given in brackets. Calibrated parameters were initially estimated using the references listed above, then manually tuned until the simulation outputs fit within the data bounds (see Sect. 3.1 for more details). Default values were used in the basic_simulation example on Epiabm’s GitHub web-page: https://github.com/SABS-R3-Epidemiology/epiabm

**Table 5:** Reference parameter set for running and updating the model (excluding household age parameters, place parameters, probability distributions, within-host progression probabilities and WAIFW matrix values: see Tables 6, 7, 8, 9, 10, 11 and 12)

Parameter description	Value	Justification
Basic reproduction number	4.3	(Blower et al. 1995)
$\mathbb {P}(\text {cavitated disease})$	0.4	(Gadkowski and Stout 2008; Zhang et al. 2016; Urbanowski et al. 2020)
Male cavitated disease probability multiplier	1.2	(Balogun et al. 2021), calibrated, see §3.1
Female cavitated disease probability multiplier	0.8	(Balogun et al. 2021), calibrated, see §3.1
Mean latent period (days)	746.863	(Borgdorff et al. 2011)
Maximum latent period (years)	12.8	(Borgdorff et al. 2011)
Mean infectious period (days)	1215.45	(Tiemersma et al. 2011)
Maximum infectious period (years)	10	(Tiemersma et al. 2011)
Mean male diagnosis delay (days)	38.5161	(Nsawotebba et al. 2025)
Mean female diagnosis delay (days)	33.3841	(Nsawotebba et al. 2025)
Mean time to stop treatment early (days)	60	assumed
Treatment period (days)	180	(Prats et al. 2016)
Mean time to relapse (days)	210	assumed
Maximum time to relapse (days)	730	(Prats et al. 2016)
Number of quantiles per state transition ICDF	20	default
Time steps per day	1	default
Maximum household size (people)	16	assumed
Proportion of females (%)	53.0192	(Uganda Bureau of Statistics 2024)
Proportion of males (%)	46.9808	(Uganda Bureau of Statistics 2024)
Initial proportion of female cases (%)	31.25	(Boum et al. 2014), calibrated, see §3.1
Initial proportion of male cases (%)	68.75	(Boum et al. 2014), calibrated, see §3.1
Place attack rate	0.506	(McIntosh et al. 2019), calibrated, see §3.1
Household attack rate	0.506	(McIntosh et al. 2019; Whalen et al. 2011), calibrated, see §3.1
$\mathbb {P}(\text {Individual is vaccinated})$	0.83	(Who/Unicef 2022)
Infectiousness decrease due to vaccination (%)	74	(Fromsa et al. 2024)
Susceptibility decrease due to vaccination (%)	20	(Roy et al. 2014)
WAIFW matrix stratified by age scalar	9.9	calibrated, see §3.1
WAIFW matrix stratified by gender scalar	0.44	calibrated, see §3.1
Natural death rate (people $\text {day}^{-1}$)	0.0000133	(United Nations 2024b)
Birth rate (people $\text {day}^{-1}$)	0.0005	(Uganda Bureau of Statistics 2024)
Married proportion (%)	56.5	(Uganda Bureau of Statistics 2024)
Polygamous marriages (%)	13.6	(Uganda Bureau of Statistics 2016)
Divorce rate ($\text {year}^{-1}$)	0.017979	assumed

Units are given in brackets. Calibrated parameters were initially estimated using the references listed above, then manually tuned until the simulation outputs fit within the data bounds (see Sect. 3.1 for more details). Default values were used in the basic_simulation example on Epiabm’s GitHub web-page: https://github.com/SABS-R3-Epidemiology/epiabm. Abbreviations: ICDF, inverse cumulative distribution function; WAIFW, Who Acquired Infection From Whom

**Table 6:** Reference parameter set for parameters governing household ages and places

Parameter description	Value	Justification
Mean child age gap (years)	3	(Uganda Bureau of Statistics 2018)
Minimum adult age (years)	10	(United Nations 2024a)
Maximum child age (years)	19	(UNESCO Institute for Statistics 2023)
Minimum parent age gap (years)	10	(United Nations 2024a)
Maximum parent age gap (years)	59	assumed
$\mathbb {P}$(one child in a two-person household)	0.432432	(Uganda Bureau of Statistics 2024; United Nations, Department of Economic and Social Affairs, Population Division 2022)
$\mathbb {P}$(two children in a three-person household)	0.700855	(Uganda Bureau of Statistics 2024; United Nations, Department of Economic and Social Affairs, Population Division 2022)
$\mathbb {P}$(household consists of one old person)	0.249	(Uganda Bureau of Statistics 2021)
$\mathbb {P}$(household consists of two old people)	0.141324	(Uganda Bureau of Statistics 2024; United Nations, Department of Economic and Social Affairs, Population Division 2022)
$\mathbb {P}$(household consists of one young person)	0.001	(Uganda Bureau of Statistics 2021)
$\mathbb {P}$(household consists of two young people)	0.000568	(Uganda Bureau of Statistics 2021, 2024; United Nations, Department of Economic and Social Affairs, Population Division 2022)
$\mathbb {P}$(only child is under five)	0.274134	(Uganda Bureau of Statistics 2024)
$\mathbb {P}$(youngest of two children is under five)	0.473119	(Uganda Bureau of Statistics 2024)
$\mathbb {P}$(youngest of 3+ children is under five)	0.617555	(Uganda Bureau of Statistics 2024)
$\mathbb {P}$(no children in a three-person household)	0.563674	(United Nations, Department of Economic and Social Affairs, Population Division 2022)
$\mathbb {P}$(one child in a four-person household)	0.590047	(United Nations, Department of Economic and Social Affairs, Population Division 2022)
$\mathbb {P}$(three children in a five-person household)	0.409953	(United Nations, Department of Economic and Social Affairs, Population Division 2022)
Maximum partner age gap with older male	10	(Kelly et al. 2003)
Maximum partner age gap with older female	0	(Kelly et al. 2003)
Older generation age gap (years)	26	(United Nations 2024a)
Oldest age for young-person household (years)	17	(Uganda Bureau of Statistics 2024)
Young and single adult age probability modifier	0	assumed
Minimum age of adults with no children (years)	55	(United Nations, Department of Economic and Social Affairs, Population Division 2022)
Minimum age for old-person household (years)	65	assumed
$\mathbb {P}$(other parent absent in 3+ person house)	0	default
Average business size	124	(Goyette 2014)
Standard deviation of business size	259	(Goyette 2014)
Average school size	453	(UNESCO Institute for Statistics 2017a)
Minimum age of school pupils (years)	6	(UNESCO Institute for Statistics 2023)
Minimum age of teachers in schools (years)	20	assumed
Minimum age of workers in workplaces (years)	15	(International Labour Organization 2021)
Maximum age of school pupils (years)	19	(UNESCO Institute for Statistics 2023)
Maximum age of teachers in schools (years)	64	assumed
Maximum age of workers in workplaces (years)	64	assumed
$\mathbb {P}(\text {Pupil-age male in school})$	0.77	(UNESCO Institute for Statistics 2017a; United Nations 2024a)
$\mathbb {P}(\text {Pupil-age female in school})$	0.7903	(UNESCO Institute for Statistics 2017a; United Nations 2024a)
$\mathbb {P}(\text {20-64 year-old works as a teacher})$	0.015	(UNESCO Institute for Statistics 2017b; United Nations 2024a)
$\mathbb {P}(\text {15-24 year-old male in workplace})$	0.742	(International Labour Organization 2021)
$\mathbb {P}(\text {15-24 year-old female in workplace})$	0.659	(International Labour Organization 2021)
$\mathbb {P}(\text {25-64 year-old male in workplace})$	0.9223	(International Labour Organization 2021; Uganda Bureau of Statistics 2024)
$\mathbb {P}(\text {25-64 year-old female in workplace})$	0.8241	(International Labour Organization 2021; Uganda Bureau of Statistics 2024)
Mean classroom size	40	(Ministry of Education & Sports 2017; UNESCO Institute for Statistics 2017a)
Mean office size	10	(Ragonnet et al. 2019)

Units are given in brackets. Default values were used in the basic_simulation example on Epiabm’s GitHub web-page: https://github.com/SABS-R3-Epidemiology/epiabm

**Table 7:** Probability distributions for state transitions in reference parameter set for parameter JSON file

Percentile	Exponential	MDD	FDD
$0{\text {th}}$	0	0	0
$5{\text {th}}$	0.0513	0.0628	0.0664
$10{\text {th}}$	0.1054	0.1264	0.1335
$15{\text {th}}$	0.1625	0.1915	0.202
$20{\text {th}}$	0.2231	0.259	0.2727
$25{\text {th}}$	0.2877	0.3299	0.3465
$30{\text {th}}$	0.3567	0.4054	0.4246
$35{\text {th}}$	0.4308	0.4868	0.5085
$40{\text {th}}$	0.5108	0.5757	0.6
$45{\text {th}}$	0.5978	0.6745	0.7018
$50{\text {th}}$	0.6931	0.7862	0.8177
$55{\text {th}}$	0.7985	0.9161	0.9527
$60{\text {th}}$	0.9163	1.0722	1.1132
$65{\text {th}}$	1.0498	1.2655	1.3051
$70{\text {th}}$	1.204	1.4997	1.5286
$75{\text {th}}$	1.3863	1.764	1.7777
$80{\text {th}}$	1.6094	2.0297	2.051
$85{\text {th}}$	1.8971	2.2637	2.3538
$90{\text {th}}$	2.3026	2.4797	2.6847
$95{\text {th}}$	2.9957	2.7268	3.0672
$100{\text {th}}$	$\frac{{\text {max}}}{{\text {mean}}}$	94.7656	109.3335

All percentiles are given as multiples of the mean. Abbreviations: MDD, male diagnosis delay; FDD, female diagnosis delay. Values for the MDD and FDD distributions were calculated using Nsawotebba et al. (2025). The $100{\text {th}}$ percentile values for the MDD and FDD distributions were based on the assumption that the maximum diagnosis delay is equal to 10 years

**Table 8:** Within-host progression probabilities

Parameter	Value	Justification
$\mathbb {P}$(Latent TB to Healthy)	0.817	(Emery et al. 2021), calibrated, see §3.1
$\mathbb {P}$(Latent TB to Active TB)	0.183	(Emery et al. 2021), calibrated, see §3.1
Male multiplier for $\mathbb {P}$(Latent TB to Active TB)	1.62	(Gao et al. 2017), calibrated, see §3.1
Female multiplier for $\mathbb {P}$(Latent TB to Active TB)	0.38	(Gao et al. 2017), calibrated, see §3.1
$\mathbb {P}$(fast TB)	0.1	(Ahmad 2011)
$\mathbb {P}$(Active TB to TB death)	0.081	(World Health Organization 2025b), calibrated see §3.1
Male multiplier for $\mathbb {P}$(Active TB to TB death)	1.333	(Jimenez-Corona et al. 2006; Fox et al. 2016), calibrated, see §3.1
Female multiplier for $\mathbb {P}$(Active TB to TB death)	0.667	(Jimenez-Corona et al. 2006; Fox et al. 2016), calibrated, see §3.1
$\mathbb {P}$(Active TB to Under Treatment)	0.919	(World Health Organization 2025b), calibrated see §3.1
$\mathbb {P}$(Under Treatment to Active TB)	0.1	(World Health Organization 2025c)
$\mathbb {P}$(Under Treatment to Recovered but could relapse)	0.9	(World Health Organization 2025c)
$\mathbb {P}$(Recovered but could relapse to Healthy)	0.9	(Lambert et al. 2003), calibrated, see §3.1
$\mathbb {P}$(Recovered but could relapse to Active TB)	0.1	(Lambert et al. 2003), calibrated, see §3.1

**Table 9:** Household size in reference parameter set for parameter JSON file

Household size	HSD
1	0.051
2	0.074
3	0.117
4	0.141
5	0.27645
6	0.176928
7	0.094361
8	0.043137
9	0.017255
10	0.006135
11	0.001963
12	0.000571
13	0.000152
14	0.000037
15	0.000009
16	0.000002

HSD, household size distribution. Household size distribution values were derived based on Uganda Bureau of Statistics (2024) and Jarosz (2021)

**Table 10:** Age distributions in reference parameter set for parameter JSON file

Age group	AGD	AID	ACD
0–4	0.1477	0.000004	0.78
5–9	0.1425	0.000553	0.53
10–14	0.1324	0.075858	0.53
15–19	0.1161	0.924142	0.54
20–24	0.0963	0.999447	0.46
25–29	0.0795	0.999996	0.29
30–34	0.0631	1	0.21
35–39	0.0528	1	0.15
40–44	0.0423	1	0.12
45–49	0.0319	1	0.07
50–54	0.0275	1	0.07
55–59	0.018	1	0.05
60–64	0.0165	1	0.05
65–69	0.0095	1	0.08
70–74	0.008	1	0.07
75+	0.0159	1	0.02

AGD, age group distribution; AID, age infectiousness distribution; ACD, age contact distribution (used for spatial transmissions).

Age group distribution values were derived based on Uganda Bureau of Statistics (2024) and rounded to 4 decimal places. The 75+ age group was further split into 75-79 (31.1860% of the 75+ age group to 4 decimal places); 80-84 (28.7061%); 85-89 (25.7229%); 90-94 (10.8811%) and 95+ (3.5039%). Age infectiousness distribution values were derived based on Ragonnet et al. (2019). Age contact distribution values were derived based on Prem et al. (2017)

**Table 11:** Who Acquired Infection From Whom (WAIFW) matrix stratified by age

	1	2	3	4	5	6	7	8	9	10	11	12	13	14	15	16
1	8.901	4.138	1.831	1.021	1.314	1.633	1.532	1.134	0.579	0.345	0.312	0.253	0.129	0.087	0.037	0.016
2	4.159	23.762	4.105	1.118	0.631	1.247	1.387	1.167	0.854	0.386	0.243	0.219	0.137	0.080	0.031	0.017
3	1.414	6.262	15.817	1.862	0.786	0.620	0.784	0.821	0.703	0.411	0.219	0.118	0.067	0.063	0.032	0.017
4	0.716	1.620	5.251	12.645	2.615	1.208	0.861	0.980	0.890	0.724	0.411	0.231	0.108	0.035	0.016	0.009
5	0.956	0.803	0.779	4.251	5.198	2.408	1.469	1.299	0.957	0.848	0.623	0.372	0.189	0.019	0.012	0.008
6	1.469	0.878	0.453	1.332	2.824	3.311	1.989	1.592	1.305	0.958	0.836	0.476	0.241	0.017	0.005	0.006
7	1.408	1.929	1.306	0.711	1.318	2.046	2.267	1.872	1.429	1.123	0.767	0.547	0.238	0.020	0.010	0.005
8	1.318	1.909	1.397	1.075	0.904	1.545	1.722	2.158	1.864	1.250	0.952	0.493	0.186	0.036	0.013	0.004
9	0.791	1.322	1.217	1.165	0.988	1.317	1.563	1.687	1.913	1.513	1.142	0.480	0.239	0.027	0.007	0.006
10	0.514	1.002	1.010	1.443	0.821	1.025	1.248	1.403	1.382	1.336	0.969	0.568	0.195	0.018	0.011	0.011
11	0.583	0.913	1.114	1.141	0.802	1.183	1.228	1.229	1.600	1.609	1.283	0.819	0.250	0.019	0.010	0.013
12	0.988	1.288	1.052	1.029	0.709	1.031	1.219	1.065	1.228	1.033	1.073	0.815	0.358	0.038	0.015	0.022
13	0.794	0.892	0.730	0.572	0.480	0.652	0.706	0.828	0.770	0.758	0.653	0.664	0.228	0.079	0.024	0.006
14	0.504	0.870	0.781	0.309	0.191	0.137	0.223	0.266	0.160	0.090	0.099	0.098	0.092	0.052	0.060	0.011
15	0.227	0.746	0.573	0.406	0.067	0.102	0.095	0.169	0.069	0.133	0.108	0.098	0.093	0.081	0.027	0.053
16	0.382	0.551	0.801	0.600	0.100	0.107	0.085	0.158	0.152	0.147	0.157	0.135	0.044	0.068	0.040	0.023

Age groups: 1, 0-4; 2, 5-9; 3, 10-14; 4, 15-19; 5, 20-24; 6, 25-29; 7, 30-34; 8, 35-39; 9, 40-44; 10, 45-49; 11, 50-54; 12, 55-59; 13, 60-64; 14, 65-69; 15, 70-74; 16, 75+. Values have been taken from Prem et al. (2017) (with justification for doing so coming from Wallinga et al. (2006)) and rounded to 3 decimal places

**Table 12:** Who Acquired Infection From Whom (WAIFW) matrix stratified by gender

Infector	Infectee	
	Female	Male
Female	0.37	0.3
Male	0.37	0.56

Values have been taken from Miller et al. (2021) (with justification for doing so coming from Wallinga et al. (2006))

## Appendix D Justification of Log-Normal Business Size Distribution Assuming Gibrat’s Law Holds

A business of size $S_t$ at time *t* (that is, one with $S_t$ employees) will have increased in size by growth rate $\frac{S_t - S_{t-1}}{S_{t-1}} = \varepsilon _t$ from its size at time t-1, $S_{t-1}$. In other words, $S_t = S_{t-1}(1 + \varepsilon _t)$. We can iteratively apply this formula to calculate the business sizes at earlier times t-1,t-2,...,1 to arrive at the formula $S_t = S_0\underbrace{(1+\varepsilon _1)\cdots (1+\varepsilon _t)}_{t \text { factors}}$, where $S_0$ is the initial business size. Taking the natural logarithm of both sides gives $\ln S_t = \ln S_0 + \ln (1 + \varepsilon _1) + \cdots +\ln (1+\varepsilon _t)$. If we make infinitely many measurements of the business size between the start time of the business and time *t*, with each measurement coming an infinitesimal amount of time after the previous measurement, then we can assume that the contribution of $\ln S_0$ to $\ln S_t$ will be negligible, and $|\varepsilon _n| < 1$, where $n \in \{1,...,t\}$. By calculating the Taylor expansion of $\ln (1+\varepsilon _n)$, we find it will approximately equal $\varepsilon _n$ for all *n*. Thus, $\ln S_t \approx \varepsilon _1 + \cdots + \varepsilon _t$. If Gibrat’s law holds, and the business growth rate is independent of its current size, then we assume that $\varepsilon _n$ is normally distributed with mean μ and variance $\sigma ^2$ for all *n*. In this case, $\ln S_t$ is approximately normally distributed with mean tμ and variance $t\sigma ^2$. Thus, $S_t$ should be approximately log-normally distributed, as expected.

## Appendix E Gamma-Distributed Latent Period

To investigate how sensitive our results were to the probability distribution used for sampling the latent period of individuals with Latent TB, we ran 300 counterfactual simulations where the latent periods were drawn from a Gamma distribution with shape parameter 2. Gamma distributions have been used to model the latent period in other tuberculosis transmission models (e.g. Jabbari et al. (2016)), by splitting up the Latent TB compartment into multiple sub-compartments, with exponentially-distributed transitions between one sub-compartment and the next. As the sum of *n* random variables drawn from an exponential distribution with rate β will be Gamma-distributed with shape *n* and rate β, this results in the overall time spent in a latent infection state being Gamma-distributed. There is evidence that latent infection occurs in two phases in humans: ‘early latency’ within the first two years post-infection with higher reactivation rates, and ‘late latency’ after that where reactivation rates are reduced (Colangeli et al. 2020). As a result, if we assume that the early latency and late latency phases are drawn from identical exponential distributions, an intuitive choice for the shape of a Gamma-distributed latent period would be 2, with the transition from early latency to late latency and late latency to active TB each being exponentially distributed.

A summary of the total case numbers and male-to-female case ratios are shown in Fig. 19. Although there is a decrease in the total case numbers during the first year under the assumption of a Gamma-distributed latent period, after that they increase and eventually catch up to the total case numbers predicted by an exponentially distributed latent period at the end of the simulated six-year period. The male-to-female case ratio is slightly higher when the latent period is Gamma-distributed throughout the simulated period, but is essentially no different to the ratio predicted with an exponentially-distributed latent period.

A Mann–Whitney U test comparing the endpoint distributions of the total case numbers from simulations with exponentially-distributed latent periods to total case numbers from simulations with Gamma-distributed latent periods suggested the two quantities came from distributions that were not significantly different (i.e. p>0.05); the same was found for the male-to-female case ratios. Therefore, we may reasonably conclude the model is robust to our choice of latent period distribution.

## Appendix F Consistency Analysis

A consistency analysis was conducted following the methodology set out by Hamis et al. (2021). This determined the number of simulations required to be sufficient so that the long-term behaviour of quantities such as averages and uncertainty intervals are consistent. By this, we mean that the differences between the averages and uncertainty intervals of the outputs of two alternative sets of simulations of the model with the same inputs should have a small statistical significance. Therefore, running this number of simulations should mitigate any intrinsic uncertainty due to the model’s stochastic nature. A brief overview of this methodology is described in the following section.

The *A*-measure of stochastic superiority (hereafter referred to as the *A*-measure) can be used to compare two distributions, *B* and *C* (either continuous or discrete), and check if they are ‘stochastically equal’ with respect to some random variable *X*. In other words, if $X_B$ and $X_C$ denote a random data sample drawn from distributions *B* and *C* respectively, then the *A*-measure determines whether $P(X_B> X_C) = P(X_C > X_B)$ (Vargha and Delaney 2000). The *A*-measure is equal to

2 $$\begin{aligned} A_{12} = P(X_1 > X_2) + 0.5P(X_1 = X_2), \end{aligned}$$

with $X_1$ and $X_2$ being samples from the two data distributions in question; if the distributions are continuous, $P(X_1 = X_2) = 0$. To estimate the *A*-measure for two discrete distributions with finite sets of data samples drawn from them, where the underlying distributions are not known, an estimate can be taken, hereafter referred to as the $\hat{A}$-measure (Hamis et al. 2021; Alden et al. 2013). For underlying discrete distributions, *B* and *C*, with *m* samples $\{b_1,...,b_m\}$ and *n* samples $\{c_1,...,c_n\}$ (respectively) of random variable *X* drawn from them, the $\hat{A}$-measure is equal to

3 $$\begin{aligned} \hat{A}_{BC}(X) = \frac{1}{mn} \sum _{i=1}^{m} \sum _{j=1}^{n} 1\!\!1_{b_i > c_j} + \frac{1\!\!1_{b_i = c_j}}{2}. \end{aligned}$$

The $\hat{A}$-measure takes values between 0 and 1: if it equals 0.5, the two distributions are estimated to be ‘stochastically equal’ with respect to *X*; if it is between 0.5 and 1, distribution *B* is estimated to be ‘stochastically greater’ than distribution *C* with respect to *X*; if it is between 0 and 0.5, distribution *B* is estimated to be ‘stochastically smaller’ than distribution *C* with respect to *X*. As we primarily want to estimate if any two distributions of the same size, *n*, are stochastically equal, it makes sense to scale the $\hat{A}$-measure so that values an equivalent distance in magnitude from 0.5, regardless of whether they are less than 0.5 or greater than 0.5, are scaled to be equivalent. For all values of *n*, the scaled $\hat{A}$-measure, $\underline{\hat{A}}_{1,k'}^n(X),k' \in \{2,...,20\}$ is then calculated for each group of 20 distributions of *n* samples as follows:

4 $$\begin{aligned} \underline{\hat{A}}_{B,C}^n(X) = \left\{ \begin{array}{ll} \hat{A}_{B,C}^n(X) & \text { if } \hat{A}_{B,C}^n(X) \ge 0.5,\\ 1-\hat{A}_{B,C}^n(X) & \text { if } \hat{A}_{B,C}^n(X) < 0.5, \end{array} \right. \end{aligned}$$

for data samples *B* and *C* of size *n* with regard to random variable *X*. The $\hat{A}$-measure in Equation (4) equals:

5 $$\begin{aligned} \hat{A}_{B,C}^n(X) = \frac{1}{n^2}\sum _{i=1}^{n}\sum _{j=1}^{n}H(b_i - c_j), \end{aligned}$$

where *H*(*x*) is the Heaviside function, defined as:

6 $$\begin{aligned} H(x) = \left\{ \begin{matrix} 1 & \text {if } x > 0 \text {,}\\ \frac{1}{2} & \text {if } x = 0 \text {,}\\ 0 & \text {if } x < 0 \text {.} \end{matrix} \right. \end{aligned}$$

For each value of *n* considered, the maximum over $k' \in \{2,...,20\}$ of the scaled $\hat{A}$-measures is determined. The minimum amount of runs recommended by the consistency analysis, $n^*$, is the smallest *n* such that $\max \limits _{k' \in \{2,...,20\}}(\underline{\hat{A}}_{1,k'}^n(X))$ is below some threshold. Based on the suggestion by Hamis, Stratiev, and Powathil, this threshold is set equal to 0.56 (Hamis et al. 2021). A scaled $\hat{A}$-measure below this threshold indicates small statistical significance in stochastic differences between samples of a given distribution size.

A total of 20×(1+5+50+100+300)=9,120 simulations were run to produce the data samples used in the consistency analysis. The outputs of the first 20 runs were grouped into 20 distributions of size 1 for each output. The outputs of the next 100 runs were grouped into 20 distributions of size 5 for each output, and so on: for each $n \in \{1,5,50,100,300\}$ and each output considered, 20 distributions of size *n* were generated. Two output random variables were analysed: the total number of TB cases at the end of each simulation, and the ratio between male TB cases and female TB cases at the end of each simulation. The consistency analysis suggested $n^* \le 100$ for the ratio, and $n^* \le 300$ for the total number of cases (see Fig. 20). From this analysis, we are confident that 300 simulations for both the calibrated results and each counterfactual scenario should mitigate stochastic effects.

## Appendix G Super-Spreaders

One of our hypotheses for why the male-to-female case ratio is potentially as high as 3-to-1 was the possibility of super-spreader events, with the high proportion of male-to-male contacts in particular, allowing a few male super-spreaders the opportunity to infect a large number of other males. Multiple previous studies have found that super-spreaders cause a large proportion of TB infections (see for example Ypma et al. (2013); McCreesh and White (2018); Melsew et al. (2019); Lee et al. (2020); Teicher (2023), and Smith et al. (2023)). As males are more likely to suffer from cavitated TB (Balogun et al. 2021), and cavitated TB tends to make cases more infectious than non-cavitated TB (Urbanowski et al. 2020), the notion of males with cavitated TB being super-spreaders to other males seemed plausible.

To investigate this, whenever a transmission event occurred that led to infection, we tracked the gender of the infector responsible for transmitting the infection, as well as whether they had cavitation or not. Subsequently, we counted the number of infections each infector caused throughout a simulation and used this to fit a distribution of number of transmissions per infector (note that an infector had to have caused at least one infection to have been tracked).

In order to characterise the role which super-spreaders play in transmission dynamics, we will fit the number of transmissions per infector to a negative binomial distribution, $\text {NB}(r,p)$, taking inspiration from the methodology of Goyal, Reeves, and Schiffer (2022) (note that this probability distribution must be zero-truncated as we only count infectors who infected at least one individual). The negative binomial distribution is equivalent to a Poisson distribution, $\text {Pois}(\lambda )$, where λ is a random variable distributed as a Gamma distribution, $\lambda \sim \Gamma (r,\frac{1-p}{p})$. We then set $r = \frac{\theta }{\rho }$ and $p = \frac{1}{1+\rho }$, where θ is equal to the mean number of transmissions per infector and ρ is the index of dispersion, equal to the variance of the Gamma distribution divided by the mean. Thus, $\lambda \sim \Gamma (\frac{\theta }{\rho },\rho )$. A brief proof of the equivalence of the negative binomial distribution and the Poisson distribution with a Gamma-distributed rate parameter is given in Appendix J.

The negative binomial distribution is a convenient distribution to fit to the data, as the value of ρ can directly indicate whether super-spreaders are causing a large proportion of infections. As the value of the shape parameter of a Gamma distribution increases (and thus the *r* parameter in a negative binomial distribution increases), the Gamma distribution tends towards a normal distribution. For the same mean number of infections, θ, a smaller index of dispersion (less than 1 and close to 0: $\rho \ll 1$) would lead to a larger shape parameter in our distribution ($\lim \limits _{\rho \rightarrow 0}\frac{\theta }{\rho } = \infty$), and would indicate that the number of individuals infected by a single infector is a random variable drawn from an approximately normal distribution with little variation. Conversely, a large index of dispersion greater than 1 (ρ>1) would lead to a small shape parameter ($\lim \limits _{\rho \rightarrow \infty } \frac{\theta }{\rho } = 0$), and so indicate that the number of infections per infector is not approximately normally distributed. Furthermore, a small shape parameter leads to a more positively skewed Gamma distribution (and thus a more positively skewed negative binomial distribution), suggesting the distribution is right-tailed: in this instance, most infectors only infect a small number of individuals, whilst a small number of infectors cause a large number of infections (i.e. the mode number of infections is less than the median, which is less than the mean).

Our results suggest that super-spreaders are responsible for a large proportion of TB infections in Kampala. When we fit the zero-truncated negative binomial distribution to infections from all infectors (who caused at least one infection) across the 300 simulations, the value of ρ that could be implied from the parameter values that provided the best fit was found to equal 36.1203 (to 4 decimal places). The large value of ρ implies a right-tailed distribution, as can be seen in Fig. 21a, and suggests we can reject the hypothesis that the number of transmissions is approximately normally distributed. To confirm this, we conducted a one-sample Kolmogorov-Smirnov test on the data, which rejected the null hypothesis that the data is normally distributed at the 1% significance level. This right-tailed distribution is also observed when we stratify infectors by gender, by cavitation status, and by both gender and cavitation status, as can be seen in Appendix K. The distribution appears to provide a good fit to the data, with a squared correlation coefficient of 0.9884. Having said this, the fitted parameters for the negative binomial distribution lie close to the zero boundary, which potentially indicates a degenerate fit, so these results should be treated with caution.

Furthermore, we analysed the proportion of transmissions caused by the top 20% of infectors (hereafter referred to as $t_{20}$); on average, this came to 77.51% (to 2 decimal places) across the 300 simulations, and ranged from 62.98% to 90.10%. This is largely in-keeping with the 80/20 rule commonly observed in diseases with super-spreaders, where 20% of infectious individuals cause approximately 80% of infections (Galvani and May 2005). This would imply the average number of transmissions per infector, when sorted by rank, should fit a power law function of the form $y = Cx^{m}$, where *y* is the average number of transmissions and *x* is the infector’s rank. We found the best-fitting power law function had parameter values C=694.2174,m=-1.0417 (to 4 decimal places), with a squared correlation coefficient of 0.9992 (see Fig. 21b for the observed average number of transmissions per rank and the expected values according to the power law function). This is very close to satisfying Zipf’s law: sorting the infectors in decreasing order of the number of transmissions they caused, the number of transmissions of the $n{\text {th}}$ ranked infector is approximately inversely proportional to *n*.

Our data also indicated that males were causing a larger average number of transmissions than females (2,688 transmissions per simulation caused by males versus 1,185 caused by females, rounded to the nearest integer) and individuals with cavitated TB caused more transmissions than those with non-cavitated TB (2,745 versus 1,128 transmissions for cavitated versus non-cavitated TB, rounded to the nearest integer). Nevertheless, we wanted to find more definitive evidence that super-spreaders were more likely to be male and have cavitated TB, so we stratified the data by gender, by cavitation status and by both gender and cavitation status, and reran the analysis. The fitted parameter values for ρ can be seen in Table 13, along with the sample means.

**Table 13:** Sample means, $\bar{x}$, and fitted parameter values for ρ in the stratified zero-truncated negative binomial distributions with $r = \frac{\theta }{\rho },p=\frac{1}{1+\rho }$

$(\bar{x},\rho )$	Male	Female	Both
Cavitated	(15.4882	(13.1421	(14.7915
	64.8355)	52.2314)	61.0581)
Not cavitated	(6.0491	(5.0275	(5.5857
	17.7214)	13.4148)	15.7373)
Both	(11.1621	(8.0748	(9.9932
	41.9759)	26.8695)	36.1203)

As the distribution is zero-truncated, the values of θ are less informative, since they do not equal the sample mean, and hence have not been shown here

**Table 14:** Mean and (minimum, maximum) values of $t_{20}$ by stratification across the 300 simulations

	Male	Female	Both
Cavitated	0.7934	0.7550	0.7968
	(0.6131, 0.9293)	(0.4500, 0.9751)	(0.6283, 0.9357)
Not cavitated	0.6794	0.6473	0.6794
	(0.5016, 0.9015)	(0.4275, 0.8853)	(0.5335, 0.8617)
Both	0.7795	0.7347	0.7751
	(0.6040, 0.9159)	(0.4874, 0.9486)	(0.6298, 0.9010)

As expected, the mean number of transmissions is greater for males, regardless of cavitation status, and for infectors with cavitated TB, regardless of gender. Furthermore, male infectors with cavitated TB have the greatest number of transmissions on average, whilst female infectors with non-cavitated TB have the fewest. However, there is evidence that super-spreaders can emerge from any combination of gender and cavitation status, as ρ>1 for all stratifications. Furthermore, the average values of $t_{20}$ were between 64% and 80% for all stratifications (see Table 14).

Having said this, the average values for $t_{20}$ were greater for males than for females, regardless of whether cavitation status was stratified for, and greater for infectors with cavitated TB than those without cavitated TB, regardless of whether gender was stratified for. So, there is some weak evidence that super-spreaders are more likely to be male and have cavitated TB, but it is clear that super-spreaders can also be female and have non-cavitated TB.

## Appendix H Choice of Scale Factor and Calibration Process

To reduce computational cost, we used a scaled-down population, in which one simulated individual was representative of 50 real individuals. Using a scaled-down population when simulating TB transmission using agent-based models is a commonly employed technique (see Bui et al. (2024) for examples) and there is evidence that using scaled-down populations can still lead to near-identical results to those obtained when using a full-scale population in agent-based models of disease transmission (Hunter and Kelleher 2022). As such, the total number of simulated agents was 2% of the population size of Kampala, Uganda as estimated in 2019 (Uganda Bureau of Statistics (2019)). It should be noted that the number of places (i.e. schools and workplaces) and households was also reduced to 2% of the number of real places and households, so the number of infection events, case numbers, and amount of individuals in each compartment should match the expected amounts one would see for a population 2% the size of Kampala’s with the same demographic characteristics. Specifically, the average values for school size, workplace size and household size were equal to the observed values of these quantities in reality, while the number of schools, number of workplaces and number of households were reduced. We also ran simulations with the number of agents equal to 5% and 10% of the population size of Kampala, Uganda, and found the total case numbers followed a similar trend to the way in which total case numbers evolved with a 2% scale factor. Furthermore, the mean male-to-female case ratios were near-identical throughout the simulated time period for all three scale factors, with the main difference being a reduction in uncertainty intervals for larger scale factors: for more details, please see Appendix I.

We parametrised the model such that the initial simulated population structure matched the observed population structure for our calibrated results. We calibrated our model to ensure our model predictions were accurate by running 40 preliminary simulations with a given set of potential parameter values and passing or failing these parameter sets by checking whether the total number of TB cases and the male-to-female case ratio fit within the bounds of our data, manually tuning our parameter values and initial compartment sizes until the averages and uncertainty intervals that were generated matched those that were observed in reality. Specifically, we were satisfied our model was calibrated once the mean number of total cases was contained within the predicted 95% uncertainty interval at the end of each simulated year, with an increase in mean total case numbers year-on-year, and the mean male-to-female case ratio was fully contained within the predicted 95% uncertainty interval throughout the simulation, based on available data for the population of Uganda (World Health Organization 2024a; Boum et al. 2014) and scaled according to our simulated population size (see Fig. 22). In essence, the steps of our manual approach follow the principles of CaliPro, a calibration method for fitting parameters to complex biological models (Joslyn et al. 2020). Our original intention was to have the 95% uncertainty intervals generated by the model contained within the predicted 95% uncertainty intervals, but we were unable to achieve this with the variability of the outputs resulting from the 2% scale factor.

It is also worth noting that Epiabm, the original model we adapted to produce EpiabmTB, has previously been shown to generate realistic household structures that reflect the desired age and household size distributions (Herriott et al. 2025).

## Appendix I Sensitivity Analysis of Population Scale Factor

To test whether our choice of scale factor was significant in distorting results, we ran 300 simulations with the number of agents equal to 5% of the size of the population of Kampala, and another 300 simulations with the number of agents equal to 10% of the population of Kampala. The total case numbers evolved with a similar trend to those observed when the number of agents equalled 2% of the population size. Furthermore, the mean male-to-female case ratios were almost identical, with the main difference being a reduction in the size of the uncertainty intervals for a larger proportion of the population size being used. We conducted Mann–Whitney U tests comparing the endpoint distributions of the male-to-female case ratios for the 2% scale factor to the 5% and 10% scale factors, respectively, and found no significant differences in the distributions these were sampled from (p>0.05 for both tests) (Figs. 23, 24).

## Appendix J Proof of Equivalence of the Negative Binomial Distribution and the Gamma-Poisson Distribution

In this section, we provide a proof of the equivalence of the negative binomial distribution with parameters $r = \frac{\theta }{\rho } > 0$ and $p = \frac{1}{1+\rho } \in [0,1]$, and the Poisson distribution with rate parameter $\lambda \in (0,\infty )$, where λ is Gamma-distributed with shape parameter $r = \frac{\theta }{\rho } > 0$ and scale parameter ρ>0. In the following proof:

- $f_{\text {Pois}(\lambda )}(k)$ is the probability mass function of the Poisson distribution with rate parameter $\lambda \in (0,\infty )$ evaluated at $k \in \mathbb {N}_0$;
- $f_{\Gamma \left( \frac{\theta }{\rho },\rho \right) }(\lambda )$ is the probability density function of the Gamma distribution with shape parameter $\frac{\theta }{\rho } > 0$ and scale parameter ρ>0 evaluated at $\lambda \in (0,\infty )$;
- $f_{\Gamma \left( \frac{\theta }{\rho }+k,\frac{\rho }{1+\rho } \right) }(\lambda )$ is the probability density function of the Gamma distribution with shape parameter $\frac{\theta }{\rho } + k > 0$ and scale parameter $\frac{\rho }{1+\rho } > 0$ evaluated at $\lambda \in (0,\infty )$;
- $f_{\text {NB} \left( \frac{\theta }{\rho },\frac{1}{1+\rho } \right) }(k)$ is the probability mass function of the negative binomial distribution with parameters $\frac{\theta }{\rho } > 0$ and $\frac{1}{1+\rho } \in [0,1]$ evaluated at $k \in \mathbb {N}_0$;
- Γ(z) is the Gamma function evaluated at z>0.

All other symbols have their standard mathematical interpretation. To prove the two distributions are equivalent, we want to show that, for any input *k*, the probability mass function of the negative binomial distribution evaluated at *k* is identical to the product of the probability mass function of the Poisson distribution with rate parameter λ evaluated at *k* and the probability density function of the Gamma distribution evaluated at λ, integrated over all possible values of λ. We begin the proof by calculating this integral.

7 $$\begin{aligned}&\int _0^\infty f_{\text {Pois}(\lambda )}(k)\times f_{\Gamma \left( \frac{\theta }{\rho },\rho \right) }(\lambda ) d\lambda \end{aligned}$$

8 $$\begin{aligned} =&\int _0^\infty \frac{\lambda ^k}{k!}\exp {(-\lambda )} \times \frac{1}{\Gamma \left( \frac{\theta }{\rho } \right) \rho ^{\frac{\theta }{\rho }}} \lambda ^{\frac{\theta }{\rho }-1} \exp {\left( -\frac{\lambda }{\rho } \right) } d\lambda \end{aligned}$$

In the above step, we substitute in the probability mass function of the given Poisson distribution and the probability density function of the given Gamma distribution. We can take the constant $\frac{1}{\rho ^{\frac{\theta }{\rho }}} \frac{1}{k!\Gamma \left( \frac{\theta }{\rho } \right) }$ outside of the integral and multiply the different powers of λ and *e* together to obtain the following.

9 $$\begin{aligned}&\int _0^\infty \frac{\lambda ^k}{k!}\exp {(-\lambda )} \times \frac{1}{\Gamma \left( \frac{\theta }{\rho } \right) \rho ^{\frac{\theta }{\rho }}} \lambda ^{\frac{\theta }{\rho }-1} \exp {\left( -\frac{\lambda }{\rho } \right) } d\lambda \end{aligned}$$

10 $$\begin{aligned} =&\frac{1}{\rho ^{\frac{\theta }{\rho }}}\frac{1}{k!\Gamma \left( \frac{\theta }{\rho } \right) } \int _0^\infty \lambda ^{\frac{\theta }{\rho } + k - 1} \exp {\left( -\lambda \frac{1 + \rho }{\rho } \right) } d\lambda \end{aligned}$$

The probability density function of the Gamma distribution with shape parameter $\frac{\theta }{\rho }+k$ and scale parameter $\frac{\rho }{1+\rho }$ evaluated at λ is equal to $\frac{1}{\Gamma \left( \frac{\theta }{\rho } + k \right) \left( \frac{\rho }{1+\rho } \right) ^{\frac{\theta }{\rho }+k}}\lambda ^{\frac{\theta }{\rho }+k-1}\exp {\left( -\lambda \frac{1+\rho }{\rho } \right) }$. We can use this fact to simplify the expression as follows.

11 $$\begin{aligned}&\frac{1}{\rho ^{\frac{\theta }{\rho }}}\frac{1}{k!\Gamma \left( \frac{\theta }{\rho } \right) } \int _0^\infty \lambda ^{\frac{\theta }{\rho } + k - 1} \exp {\left( -\lambda \frac{1 + \rho }{\rho } \right) } d\lambda \end{aligned}$$

12 $$\begin{aligned} =&\frac{1}{\rho ^{\frac{\theta }{\rho }}}\frac{1}{k!\Gamma \left( \frac{\theta }{\rho } \right) }\Gamma \left( \frac{\theta }{\rho }+k \right) \left( \frac{\rho }{1 + \rho } \right) ^{\frac{\theta }{\rho } + k} \int _0^\infty f_{\Gamma \left( \frac{\theta }{\rho } + k,\frac{\rho }{1 + \rho } \right) }(\lambda ) d\lambda \end{aligned}$$

A probability density function must integrate to 1 over its support, so we can determine that the integral is equal to 1 and subsequently remove it, then simplify the expression to arrive at the following.

13 $$\begin{aligned}&\frac{1}{\rho ^{\frac{\theta }{\rho }}}\frac{1}{k!\Gamma \left( \frac{\theta }{\rho } \right) }\Gamma \left( \frac{\theta }{\rho }+k \right) \left( \frac{\rho }{1 + \rho } \right) ^{\frac{\theta }{\rho } + k} \int _0^\infty f_{\Gamma \left( \frac{\theta }{\rho } + k,\frac{\rho }{1 + \rho } \right) }(\lambda ) d\lambda \end{aligned}$$

14 $$\begin{aligned} =&\frac{\Gamma \left( \frac{\theta }{\rho } + k \right) }{k! \Gamma \left( \frac{\theta }{\rho } \right) } \left( \frac{\rho }{1 + \rho } \right) ^k \left( \frac{1}{1 + \rho } \right) ^{\frac{\theta }{\rho }} \end{aligned}$$

The function we are left with is equal to the probability mass function of the negative binomial distribution we were aiming to prove was equivalent to the integral stated at the start of the proof (as $\frac{\rho }{1+\rho } = 1 - \frac{1}{1+\rho }$).

15 $$\begin{aligned}&\frac{\Gamma \left( \frac{\theta }{\rho } + k \right) }{k! \Gamma \left( \frac{\theta }{\rho } \right) } \left( \frac{\rho }{1 + \rho } \right) ^k \left( \frac{1}{1 + \rho } \right) ^{\frac{\theta }{\rho }}\end{aligned}$$

16 $$\begin{aligned} =&f_{\text {NB} \left( \frac{\theta }{\rho },\frac{1}{1 + \rho } \right) }(k) \end{aligned}$$

Hence, our proof is complete.

## Appendix K Super-Spreaders by Stratification

See Figs. 25, 26 and 27.

## Footnotes

1 It should be noted that (Boum et al. 2014) was a calibration target, so we have been able to achieve in-sample agreement rather than independent validation.
